# Efficient genome editing of an extreme thermophile, *Thermus thermophilus,* using a thermostable Cas9 variant

**DOI:** 10.1038/s41598-021-89029-2

**Published:** 2021-05-05

**Authors:** Bjorn Thor Adalsteinsson, Thordis Kristjansdottir, William Merre, Alexandra Helleux, Julia Dusaucy, Mathilde Tourigny, Olafur Fridjonsson, Gudmundur Oli Hreggvidsson

**Affiliations:** 1 Matís, Reykjavík, Iceland; 2 University of Iceland, Reykjavík, Iceland; 3 Aix-Marseille Université, Marseille, France; 4 Université de Strasbourg, Strasbourg, France; 5 Université Pierre et Marie Curie, Paris, France

**Keywords:** CRISPR-Cas9 genome editing, Microbiology

## Abstract

Thermophilic organisms are extensively studied in industrial biotechnology, for exploration of the limits of life, and in other contexts. Their optimal growth at high temperatures presents a challenge for the development of genetic tools for their genome editing, since genetic markers and selection substrates are often thermolabile. We sought to develop a thermostable CRISPR-Cas9 based system for genome editing of thermophiles. We identified CaldoCas9 and designed an associated guide RNA and showed that the pair have targetable nuclease activity in vitro at temperatures up to 65 °C. We performed a detailed characterization of the protospacer adjacent motif specificity of CaldoCas9, which revealed a preference for 5′-NNNNGNMA. We constructed a plasmid vector for the delivery and use of the CaldoCas9 based genome editing system in the extreme thermophile *Thermus thermophilus* at 65 °C*.* Using the vector, we generated gene knock-out mutants of *T. thermophilus*, targeting genes on the bacterial chromosome and megaplasmid. Mutants were obtained at a frequency of about 90%. We demonstrated that the vector can be cured from mutants for a subsequent round of genome editing. CRISPR-Cas9 based genome editing has not been reported previously in the extreme thermophile *T. thermophilus.* These results may facilitate development of genome editing tools for other extreme thermophiles and to that end, the vector has been made available via the plasmid repository Addgene.

## Introduction

CRISPR-Cas systems are adaptive immune systems in bacteria and archaea that defend against invading genetic materials such as viruses and plasmids^1,2^. They are composed of a CRISPR array and *cas* genes. The CRISPR array contains a collection of short sequences called spacers, that are complimentary to foreign DNA/RNA sequences called protospacers. A repeat sequence separates the spacers^3,4^. The CRISPR array is transcribed into RNA and processed into smaller fragments, mature crRNAs, each containing a single spacer^5^. Upon invasion of foreign nucleic acid into a prokaryotic cell, Cas nucleases are guided by crRNAs to a complimentary protospacer where they cleave the DNA or RNA^2^. CRISPR-Cas systems are classified into types I–VI on basis of the effector proteins involved^6,7^. In type II systems (CRISPR-Cas9), Cas9 mediates crRNA:protospacer interactions and nuclease activity, causing DNA double strand breaks^8^. An RNA transcript emanating from type II CRISPR loci, termed trans-activating RNA (tracrRNA) forms a duplex with crRNA to guide Cas9^9^. In addition to crRNA:protospacer base pairing, Cas9 nuclease activity is dependent on a sequence motif directly adjacent to the targeted protospacer—a protospacer adjacent motif, PAM^10–12^.

Type II CRISPR-Cas systems have been adapted as genome editing tools for a wide variety of organisms. The most widely used system originates from *Streptococcus pyogenes* and consists of its Cas9 enzyme (SpyCas9), crRNA and tracrRNA—or more frequently a fused variant of the latter two called guide RNA (gRNA)^8^. The spacer sequence of crRNA or gRNA can be engineered to target any genomic sequence, but SpyCas9 only cleaves DNA adjacent to the PAM sequence 5′-NGG^8^. Engineered variants of SpyCas9 and other Cas9 enzymes have different PAM specificities^13^, allowing further targeting possibilities. After delivery of the system into a cell the Cas9:gRNA complex binds to the targeted sequence and causes a double strand break in the DNA. If left unrepaired, the double strand break is lethal to cells. In mammalian cells the resulting DNA ends can be ‘fused’ together by an error-prone DNA repair mechanism called non-homologous end joining, which frequently results in insertion/deletion mutations. As a result, generation of knock-out mutations in mammalian cells can be achieved by introducing only Cas9 and gRNA into the cells. Non-homologous end joining repair pathways are not universally present in prokaryotes^14^. When the system is utilized for genome editing in bacteria a homologous recombination (HR) construct—which can be designed to achieve genomic deletions, insertions or substitutions—is therefore generally supplied with the system for directing genetic modifications via homology directed repair^15^ and Cas9:gRNA acts as a selection system by eliminating non-edited clones^16^ (supplementary file 1). The success of Cas9 based genome editing in bacteria is therefore dependent on the activity of Cas9; the activity of DNA repair mechanisms in the host; and on properties of the DNA repair template (its size, whether it is linear or circular, etc.). High Cas9 activity contributes to low transformation efficiency (high lethality) and high genome editing efficiency, while high activity of DNA repair contributes to increased transformation efficiency and increased genome editing efficiency. To achieve the right balance between editing and transformation efficiencies, it may be necessary to modulate the activity of Cas9 by choosing a weak, strong or inducible promoter, as appropriate, and to increase the activity of DNA repair by supplying exogenous recombinases.

SpyCas9 is not stable in vivo beyond 42 °C, and therefore not applicable for genome editing in extreme thermophiles^17^. Recently, thermostable Cas9 enzymes—designated GeoCas9, ThermoCas9, AceCas9, IgnaviCas9 and NsaCas9—have been identified and characterized^18–22^. In vitro assays suggest they are active at temperatures of at least 60–70 °C. Knock-out of two genes was attempted separately with ThermoCas9 in the moderate thermophile *Bacillus smithii* at 55 °C^18^. Application of the system resulted in complete lethality for one of the targeted genes, and predominantly in generation of clones of a mixed WT/KO (wild-type/knock-out) genotype for the other. Perhaps inclusion of an exogenous recombinase would facilitate more efficient genome editing with ThermoCas9 in *B. smithii.* Expression of ThermoCas9 and AceCas9 in combination with gRNAs targeting genomic loci in the moderate thermophile *Clostridium thermocellum* resulted in no or limited cell death, and therefore neither was applicable for genome editing applications^23^. The underlying reason for the observed lack of Cas9 activity was however not explored and it therefore cannot be ruled out that ThermoCas9 and/or AceCas9 could be utilized in *C. thermocellum* genome editing, e.g., if controlled with a suitable promoter. In *C. thermocellum* GeoCas9 was however successfully used for genome editing, yielding a high proportion of desired KO clones when used in combination with a recombinase and grown at 50–55 °C^23^. CRISPR-Cas9 based genome editing was recently achieved in cells growing at 65 °C in the extreme thermophile *Thermoanaerobacter ethanolicus* at very low efficiency^24^, i.e., yielding predominantly WT or mixed genotype clones rather than the desired KO clones. Efficient genome editing using thermostable CRISPR-Cas9 systems has not been demonstrated in extreme thermophiles. Where tested, it remains unclear whether inefficiency of the system is the result of instability of Cas9 at high temperatures, if it is the result of inefficient genome repair in the cellular system tested, or for other reasons.

Today, the favoured model chassis species for systems and synthetic biology efforts, e.g. *Escherichia coli, Saccharomyces cerevisiae*, and *Bacillus* spp., are derived from a narrow environmental range regarding temperature and pH. They are the organisms of choice as chassis species for metabolic engineering due to their genetic tractability and experimentally supported vast knowledge on various aspects of their biology^25^. However, they are not always the best potential species for industrial applications. Their substrate range may be limited relative to feedstock, and key properties may be missing for coping with physico-chemical conditions favourable e.g. for exploiting raw and recalcitrant 2nd and 3rd generation feedstocks^26^. Still, the work on these organisms is generating the important fundamental groundwork in the field of metabolic engineering and paving the way for other organisms.

Extreme thermophilic heterotrophic bacteria are highly interesting from an industrial processing perspective. The ability to grow at high temperatures in bioreactors reduces costs of cooling and prevents contamination by mesophilic spoilage bacteria. High temperature also increases solubility of polysaccharides and consequently leads to reduced viscosity of fermentation broths. This may alleviate, to significant extent, scale up problems of mixing and aeration and enable greater substrate loadings. Elevated temperatures can also enable more cost-effective recovery of volatile products by distillation (preferably continuous) or gas stripping that would reduce inhibition of products, therefore, prolonging the fermenting life of cultures and resulting in increased yields^26,27^. Despite their potential, no examples of industrial microbial production using thermophilic organisms exist on a commercial scale. Industrial bioprocessing using mesophilic microorganisms have however been a reality for decades, of which ethanol production from starch rich feedstocks using yeast is by far the most commercially successful^28^.

Genetic tools for genome editing are well established for many mesophiles, but to a much lesser degree for thermophiles^28^. Depending on species, this may include lack of genetic transfer systems, genomes being poorly/not sequenced, and particularly due to a lack of selectable markers. When recyclable marker systems are available, their application for genome engineering is generally time consuming. More efficient genome editing tools may therefore contribute to increased practical application of thermophilic microorganisms. In this paper we describe the development of a CRISPR-Cas9 system for genome editing of extremely thermophilic bacteria. We utilized a Cas9 enzyme closely related to ThermoCas9 and GeoCas9 that exhibited activity at high temperatures in vitro; we characterized its PAM sequence in detail; and demonstrated its function as a genome editing tool in vivo at 65 °C in the model extreme thermophilic species *Thermus thermophilus.* These results may facilitate development of genome editing tools for other extreme thermophiles and to that end we have made a vector carrying the system available to other researchers through the plasmid repository Addgene (plasmid #164264).

## Results

### Identification of CaldoCas9

**Figure 1:**
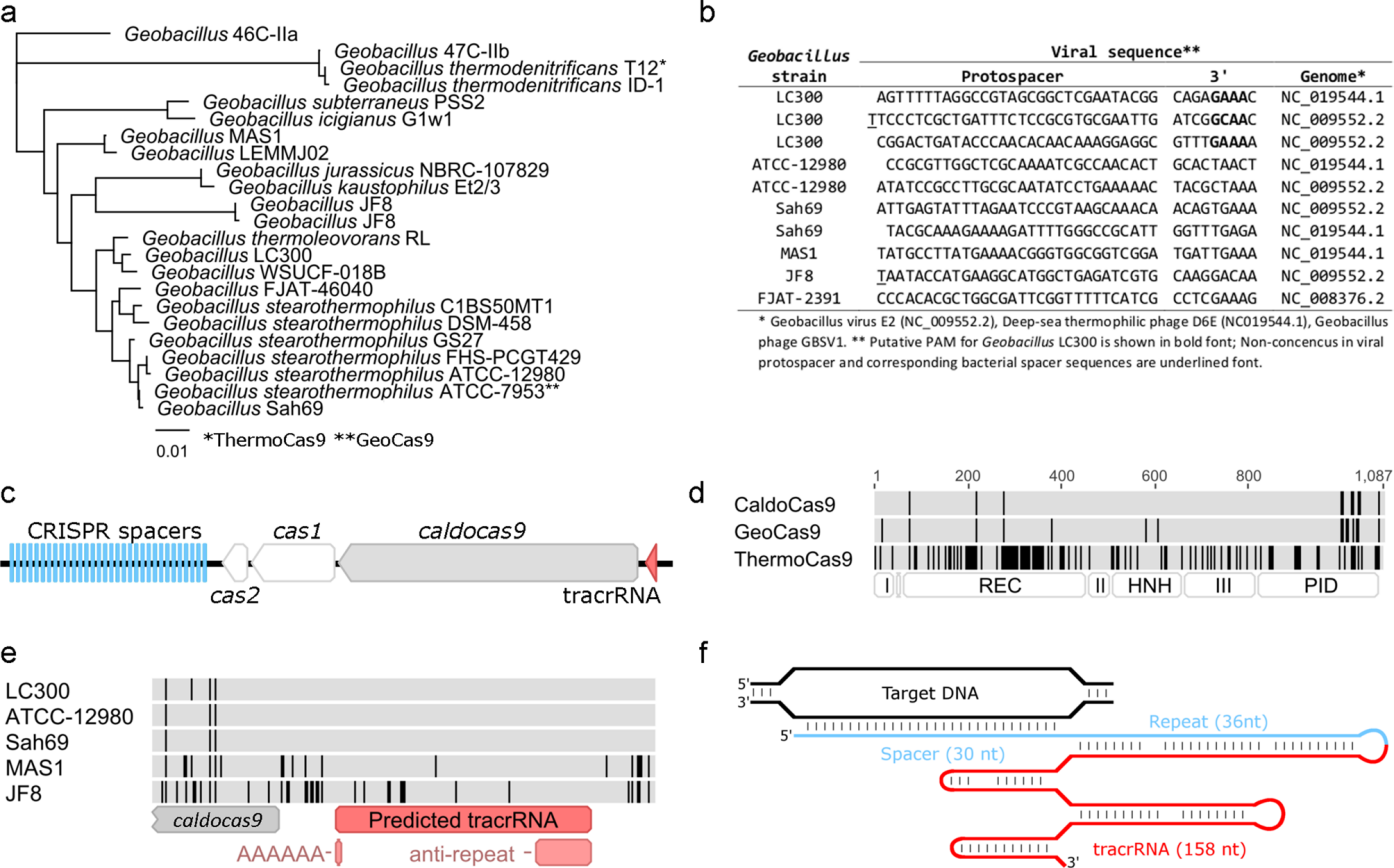
Identification of CaldoCas9 and design of gRNA. (**A**) Phylogeny of Cas9 enzymes identified in publicly available genomes of *Geobacillus* strains. Respective strain names are indicated. Scale bar indicates average number of amino acid residue substitutions per site. (**B**) A list of protospacers identified in viral genomes that are complimentary to spacers extracted from *Geobacillus* strains. Protospacer sequences and 9 bases downstream of the protospacer are shown. (**C**) A schematic illustrating organization of the CRISPR-Cas9 genomic region of *Geobacillus* strain LC300. The putative location of the tracrRNA is indicated, and of genes encoding Cas1, Cas2, and CaldoCas9. (**D**) Alignment of amino acid residue sequence of CaldoCas9 with the previously characterized enzymes GeoCas9 and ThermoCas9. Scale indicates polypeptide length. Each residue per enzyme is indicated in gray or black color, where gray indicates sequence consensus in all three enzymes, and black indicates non-consensus. At the bottom of the schematic, locations of Cas9 domains are indicated. (**E**) Genome sequence alignment of five *Geobacillus* strains, showing an approximately 0.3 kb region upstream of the *caldocas9* open reading frame. Each nucleotide is indicated in gray or black color, where gray indicates consensus between sequences and black indicates non-consensus. Location of an CRISPR anti-repeat sequence, a poly-A sequence, and putative tracrRNA are indicated. (**F**) A schematic illustration of the designed gRNA. Predicted base-pairing is indicated with vertical black bars. The gRNA spans the entire sequence indicated in blue and red.

We sought to identify and test a thermostable CRISPR-Cas9 system in vitro for subsequent adaptation as an in vivo genome editing tool. At the time, characterization of thermostable Cas9 enzymes had not been reported and we therefore aimed to identify a previously uncharacterized Cas9 enzyme, crRNA and tracrRNA in a genome of a thermophilic prokaryote. Makarova et al. reported a gene encoding Cas9 in the genome of the thermophile *Geobacillus* sp. strain JF8 (ref 6). Since many species of the *Geobacillus* genus are thermophilic we downloaded sequences of all publicly available *Geobacillus* genomes and identified homologs of the Cas9 gene from *Geobacillus* sp. strain JF8 by nucleotide BLAST. At the time of initial analysis, we identified a total of 16 Cas9 genes in *Geobacillus* genomes, including strain JF8 (supplementary file 2). Currently, using blastp and searching NCBI’s databases restricted to *Geobacillus* genomes, 23 full length Cas9 enzymes can be identified (supplementary file 3), including ThermoCas9 and GeoCas9 (Fig. 1A). For each of the 16 Cas9 genes initially identified, we extracted all CRISPR spacers from the respective bacterial strain genome. Most genomes contained a single CRISPR array, the number of spacers ranged between 4 and 54 per array, and in total 300 spacers were identified (data not shown). We performed a BLAST search for protospacers matching the 300 spacers in all publicly available viral genomes (n = 7807, NCBI). We identified 10 protospacers complimentary to spacers from 6 different *Geobacillus* spp. strains (Fig. 1B), all from CRISPR arrays proximal to Cas9 genes. Three of the ten protospacers were complimentary to spacers in the genome of *Geobacillus* sp. strain LC300. The number of identified protospacers allowed us to define a putative PAM specificity for the *Geobacillus* sp. LC300 Cas9 enzyme: 5′-NNNNGNAA (Fig. 1B). *Geobacillus* sp. LC300 optimal growth temperature is 72 °C^29^ and thus its CRISPR-Cas9 system presented a good candidate for adaptation to a thermostable CRISPR-Cas9 genome editing tool. The CRISPR-Cas locus in *Geobacillus* sp. LC300 (Fig. 1C) contains genes *cas9, cas2* and *cas1,* and a CRISPR array with 32 spacers separated by the repeat sequence 5′-GTCATAGTTCCCCTGAGATTATCGCTGTGGTATAAT^29^. Signature genes *csn2* and *cas4* are not present in the locus, and the system is therefore of CRISPR-Cas type IIC^6^. The Cas9 enzyme of *Geobacillus* sp. LC300, which we refer to as CaldoCas9, is composed of 1087 amino acids and is therefore considerably shorter than SpyCas9 (1368 amino acids). CaldoCas9 shares a very high sequence similarity with GeoCas9 (98% identical sites) but is more distantly related to ThermoCas9 (Fig. 1A,D and supplementary file 3). CaldoCas9 and GeoCas9 sequences differ primarily in their PAM interacting domains (PID). A fusion protein has previously been reported where the GeoCas9 PID was substituted with that of CaldoCas9^19^. A preliminary investigation of the PAM sequence specificity of the fusion protein suggested preference for G and A in the fifth and eight nucleotides downstream of a target sequence, respectively. Characterization of the native CaldoCas9 enzyme has not been previously reported in vitro or in vivo*.*

### Identification of CaldoCas9 associated tracrRNA and gRNA design

Transcription data from *Geobacillus* sp. LC300 was not available, and we therefore assumed that the mature crRNA contains a full-length repeat and spacer sequence, 36 and 30 nt respectively. Using a method based on Briner et al.^30^, we identified a putative tracrRNA (Fig. 1C). First, an anti-repeat sequence, partially homologous to the CRISPR repeat, was identified in a non-coding region close to the *cas9* transcription start site (Fig. 1E) with a local nucleotide blast search within the CRISPR locus. Then we predicted the length of the tracrRNA molecule by a combination of genomic sequence conservation between different *Geobacillus* strains, identification of a potential Rho-independent transcription terminator, and in silico prediction of the RNA folding of putative tracrRNA molecules of different lengths at 72 °C. A stable crRNA:tracrRNA duplex was predicted when the tracrRNA was 158 nt (transcribed from position 2.247.840–2.247.997 in *Geobacillus* LC300 genome assembly CP008903). The predicted structure contained conserved modules of crRNA:tracrRNA duplexes^30^, i.e., a stem, nexus and two hairpins (supplementary file 4). Further it is contained within a conserved genomic region flanked by more divergent sequences, suggesting functional importance (Fig. 1E). Finally, the putative gene terminates with a poly-A sequence (Fig. 1E) following a hairpin (supplementary file 4), which are hallmarks of rho independent terminators^31^. To facilitate its use as a genome editing tool in combination with CaldoCas9, we designed a single guide RNA (gRNA, Fig. 1F). The predicted structure of the crRNA:tracrRNA suggested that the 3′ end of the crRNA and 5′ end of the tracrRNA contained three non-complimentary base-pairs (supplementary file 4). In designing the gRNA, we therefore reasoned that addition of non-complimentary bases (as described for spyCas9 by Jinek et al.^8^) was unnecessary and instead we fused the crRNA 3′-end and the tracrRNA 5′-end without addition of bases (Fig. 1F). Spacers in the genome of *Geobacillus* sp. LC300 are generally 30 bp (data not shown). Though they are presumably shortened during crRNA maturation, as observed in *Geobacillus stearothermophilus*^19^ and other prokaryotes, we chose the full length 30 nt spacer as a starting point for characterization of CaldoCas9. The gRNA we designed is therefore composed of a 30 nt spacer, 36 nt CRISPR repeat and 158 nt tracrRNA sequences, totaling 224 nt (Fig. 1F). Harrington et al. recently reported that varying stem length in the gRNA for GeoCas9 between 16 and 36 nt, and tracrRNA length between 91 and 157 nt had no effect on enzymatic activity^19^.

### CaldoCas9 is a targetable nuclease and is thermostable in vitro

**Figure 2:**
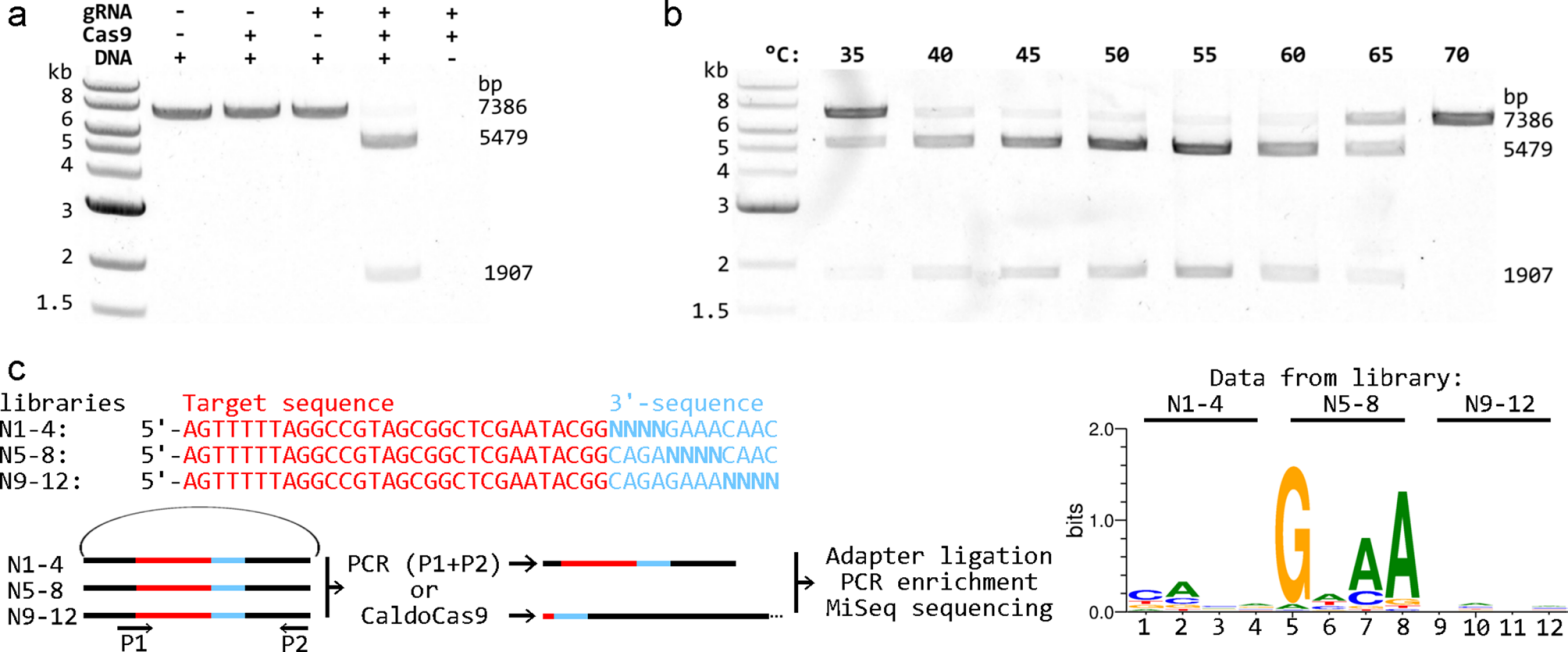
In vitro characterization of CaldoCas9 activity, thermostability and PAM specificity. (**A**) CaldoCas9, a DNA ‘target’ and gRNA containing a spacer complimentary to the target DNA were incubated in various combinations (top, +  = with, – = without) at 55 °C and the products separated on an agarose gel. Scale on left corresponds to the DNA ladder in the first well of the gel. On far right are indicated the expected band sizes of the uncut (7386 bp) and cut (5479 and 1907 bp) DNA. (**B**) CaldoCas9, a DNA ‘target’ and gRNA containing a spacer complimentary to the target DNA were incubated together at various temperatures (top) and the products separated on an agarose gel. (**C**) Top-left: three DNA libraries were prepared that contained a ‘target sequence’ that was common to all three libraries (red font) and a partially randomized sequence (3′- sequence, in blue font, randomized bases indicated as N) downstream of the target sequence. Bottom-left: the DNA libraries (carried on a circular plasmid backbone) were separately PCR amplified (primers indicated as P1 and P2) and incubated with CaldoCas9 and gRNA. The products of each were adapter ligated, PCR enriched and sequenced. Right: PAM sequences permissible for CaldoCas9 cleavage, as revealed by the experiment. The agarose gel images (**A**,**B**) have been digitally manipulated for clarity. The original images are provided in supplementary file 5.

We tested nuclease activity of CaldoCas9 and the gRNA we designed with an in vitro DNA cleavage assay. A synthesized, codon optimized gene encoding CaldoCas9 was cloned into an expression vector in frame with additional sequences to generate a fusion protein, MBP-SMT3-CaldoCas9, (MBP is maltose/maltodextrin-binding periplasmic protein and SMT3 is Ubiquitin-like protein SMT3), expressed in *E. coli* and the protein affinity column-purified. The fusion protein was used for in vitro characterization of CaldoCas9, without removal of the MBP-SMT3 sequences. We used a linearized plasmid as a target for Cas9 mediated cleavage and targeted a 30 bp sequence upstream of 5′-CAGAGAAA, consistent with the putative PAM 5′-NNNNGNAA. A gRNA containing a 30 nt spacer complimentary to this target was transcribed in vitro*.* We mixed CaldoCas9, gRNA and target DNA in different combinations and incubated at 55 °C for an hour. Combination of all three components resulted in DNA cleavage (Fig. 2A). The sizes of the DNA fragments were consistent with cleavage at the targeted site, suggesting targeted/specific endonuclease activity, a hallmark of Cas9 enzymes. We repeated the experiment using a gRNA containing a spacer that was not complimentary to the target DNA (a non-target gRNA), and observed no nuclease activity, as expected (supplementary file 5).

To investigate the thermostability of CaldoCas9 we performed further DNA cleavage assays at a range of temperatures. We used the same linearized plasmid and gRNA as before. Components of the reaction were pre-incubated separately at temperatures between 35 and 70 °C, with one mix containing CaldoCas9 and gRNA and the other containing the target DNA. After pre-incubation the components were mixed and incubation continued for 1 h. We observed cleavage of DNA at temperatures between 35 and 65 °C (Fig. 2B). No activity was detected at 70 °C and the activity was considerably reduced at 35 °C. Similarly, GeoCas9 and ThermoCas9 showed upper activity limits around 65 °C^18,19^. These data suggest that CaldoCas9 is thermostable and active in vitro at a wide temperature range, approximately between 35 and 65 °C. It is therefore a good candidate for adaptation for in vivo genome editing in thermophiles and could potentially be used for the same purpose in mesophiles.

### CaldoCas9 has a unique PAM sequence

To maximize the application potential of Cas9 enzymes for genome editing, it is important to characterize their PAM preferences in detail. We sought to achieve this for CaldoCas9 by assaying its nuclease activity against DNA libraries containing randomized protospacer adjacent sequences, a method described by Karvelis et al.^32^. Three plasmid libraries were constructed containing the same target sequence for Cas9 cleavage. Bases 1–4, 5–8 or 9–12 downstream of the target were randomized such that the sequence following (3′-sequence) was 5′-NNNNGAAACAAC for library N1-4, 5′-CAGANNNNCAAC for library N5-8, and 5´-CAGAGAAANNNN for library N9-12 (Fig. 2C, top-left). A gRNA was synthesized containing a 30 nt spacer complimentary to the target sequence. The gRNA and CaldoCas9 were incubated with each of the libraries. PAM sequences permissible for Cas9 DNA double strand break were distinguished from the rest of the respective plasmid libraries by their linearization by CaldoCas9:gRNA. Linear DNA was ‘fished’ out of the reaction by adapter ligation and subsequent PCR enrichment. MiSeq sequencing was used to reveal the sequence composition of the linearized libraries. To confirm random distribution of bases in the 3′-sequence of undigested libraries, they were PCR amplified (primers P1 and P2, Fig. 2C) and sequenced. The frequency of each base in the digested library was normalized to the frequency in the undigested library (supplementary file 6). The analysis revealed that CaldoCas9 has a strong preference for G in position 5, and for A in positions 7 and 8, downstream of the target (Fig. 2C, right). In position 7, a C base is also ‘permissible’ for DNA cleavage. These data suggest that the PAM sequence of CaldoCas9 is 5′-NNNNGNMA. This adds more detail, but is otherwise consistent with the putative PAM sequence, as suggested by protospacer search in viral genomes observed here (Fig. 1B) and reported previously^19^. The sequencing data additionally suggested that CaldoCas9 nuclease activity causes DNA double strand break between the 3^rd^ and 4^th^ bases in the protospacer at its 3′ end (data not shown).

### pTTCC allows for simple construction and delivery of CaldoCas9 based genome editing system

We sought to test the applicability of CaldoCas9 and its associated gRNA for genome editing in thermophilic bacteria. As a proof of concept, we sought to edit genes in the genome of the extreme thermophile *Thermus thermophilus. T. thermophilus* is a Gram negative, aerobic bacterium that grows at temperatures up to 85 °C^33,34^. It can be easily grown under laboratory conditions, readily takes up DNA from the environment^35,36^ and its genome sequence is published^37^. Combined with an efficient homologous recombination machinery^35,38^, these properties make for a good candidate to test the CaldoCas9 based genome editing system in vivo.

**Figure 3:**
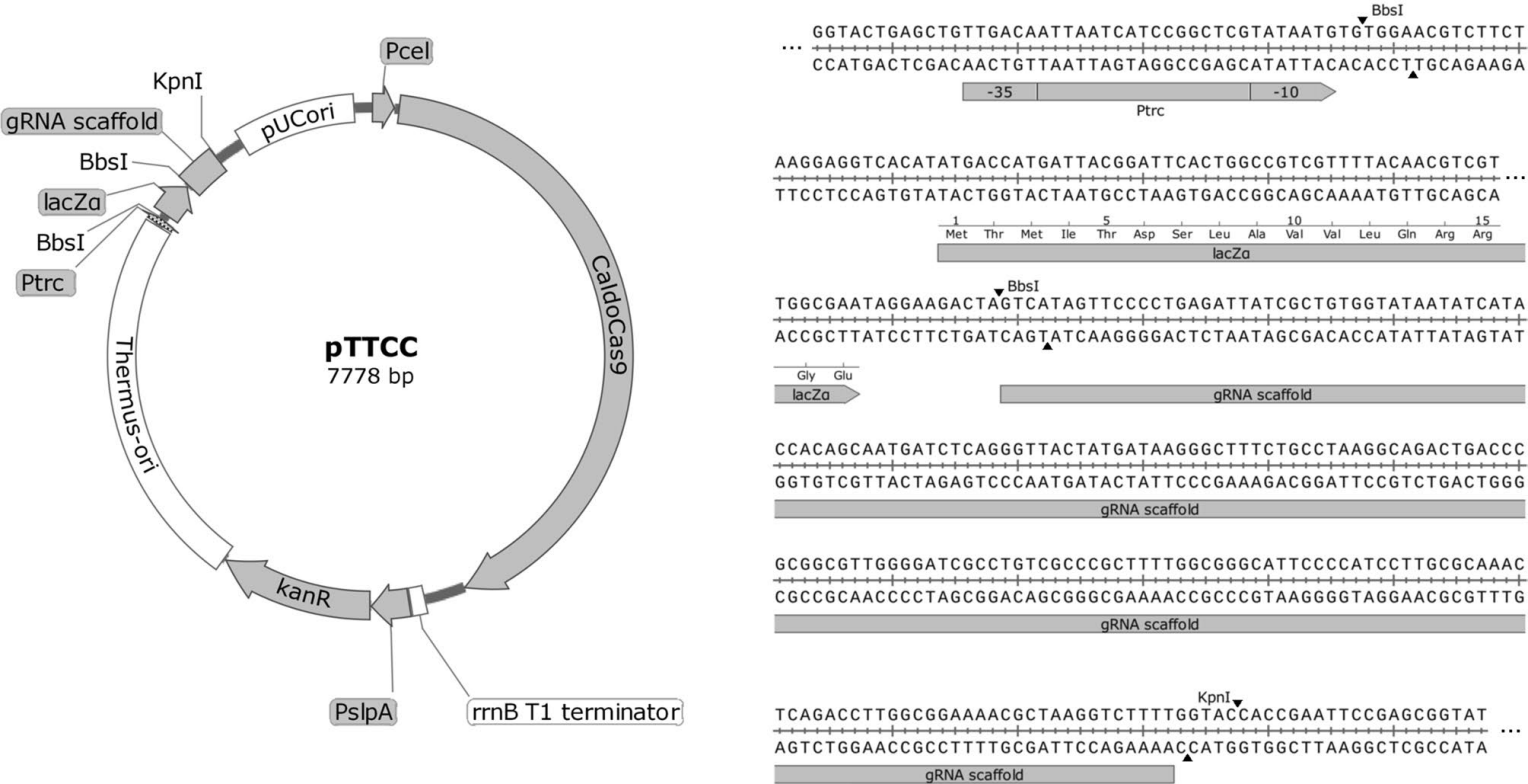
Composition of pTTCC, a CaldoCas9 genome editing system delivery vector. Left: a plasmid map illustrating the composition of pTTCC. The vector contains a gene encoding CaldoCas9 regulated by a cellobiose inducible promoter, P_cel_, and a transcription terminator; a gene encoding a thermostable enzyme conferring kanamycin resistance under regulation of a constitutive promoter P_slpA_; *T. Thermophilus* and *E. coli* specific origins of replication; and a construct for cloning gRNA spacers and homology arms for directing gene editing—shown in detail in right panel. Right: pTTCC is constructed such that the vector can be digested by *Bbs*I restriction endonuclease and spacer sequences inserted as annealed oligos via DNA ligation. The *Bbs*I restriction sites flank a gene encoding the LacZα fragment of β-galactosidase and spacer cloning can therefore be combined with blue-white screening. A unique *Kpn*I restriction site can be used for cloning of homologous recombination constructs.

We constructed a plasmid delivery system for CaldoCas9 based genome editing in *T. thermophilus* strain HB27, and have made it available through Addgene (plasmid # #164264). The plasmid, called pTTCC (plasmid *Thermus thermophilus* CRISPR-Cas9) contains (Fig. 3): a high-GC codon optimized gene encoding CaldoCas9 (supplementary file 7) under control of a cellobiose inducible promoter P_cel_ and a transcription terminator sequence; origins of replication for maintenance in *T. thermophilus* and *E. coli* (Thermus-ori, pUCori); a gene conferring kanamycin resistance (*kanR*) under control of the constitutive promoter P_slpA_; and a gRNA construct downstream of the P_trc_ promoter^39^. The plasmid was designed such that spacer sequences can be cloned as annealed oligonucleotides containing unique 4-bp overhangs on each end, into *Bbs*I restricted pTTCC (Fig. 3). Selection of clones containing the desired spacer can be selected by blue-white screening in *E. coli* strains of appropriate genotype, as the *Bbs*I sites flank a gene encoding the lacZα fragment of β-galactosidase. A unique *Kpn*I restriction site can be used for insertion of HR constructs by DNA assembly or restriction-ligation (Fig. 3).

### CaldoCas9 genome editing is highly efficient in vivo at 65 °C

We aimed to test the CaldoCas9 genome editing system, and the associated plasmid delivery system pTTCC, by knocking out genes *crtI* and *purA* in *T. thermophilus.* The genome of *T. thermophilus* strain HB27^40^ is composed of a 1.9 Mb chromosome and a 0.2 Mb megaplasmid with a GC content of 69%^37^. The bacterium is polyploid, containing an estimated 4–5 copies of the chromosome and megaplasmid each^41,42^. Genes encoding enzymes of a carotenoid biosynthesis pathway are located on the megaplasmid, making the cells yellow-pigmented. The *crtI* gene, which encodes a phytoene desaturase, converts colorless phytoene to lycopene early in the carotenoid biosynthesis pathway, and *crtI* knock-out mutants therefore appear white^43^. Cells are viable without the megaplasmid, but considerably growth retarded^41^. The *purA* gene, which encodes adenylosuccinate synthase, is located on the chromosome and is essential in adenine biosynthesis. *purA* knock-out mutants are therefore expected to require adenine supplementation for growth. The *crtI* and *purA* genes are convenient targets for testing the genome editing system, as their knock-out results in striking phenotypes that can be readily identified: WT cells are yellow and *crtI* KO mutants white; WT cells grow in media without adenine, while *purA* KO mutants do not.

**Figure 4:**
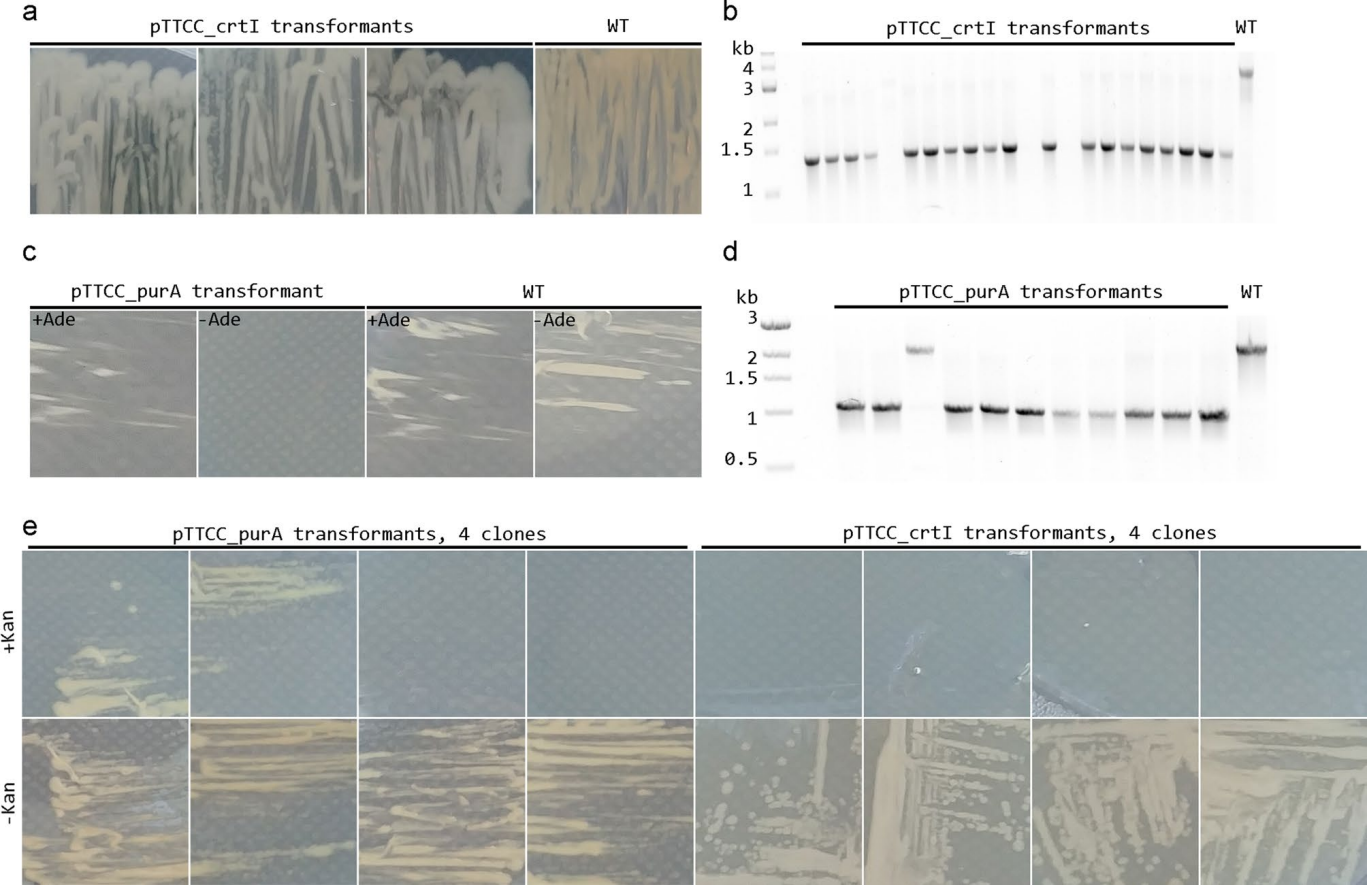
CaldoCas9 based genome editing of *T. thermophilus* at 65 °C. (**A**) pTTCC_crtI transformants streaked out on fresh media and grown overnight to reveal pigmentation difference in the transformants and WT cells. (**B**) Agarose gel electrophoresis of products from colony PCR amplification of the genomic locus containing crtI in *T. thermophilus* pTTCC_crtI transformants. Expected band size of the KO allele was 1351 bp and of WT allele 2891 bp. (**C**) pTTCC_purA transformants streaked out on minimal media with and without adenine supplementation (+ Ade − Ade, respectively) to reveal the adenine auxotrophic phenotype. (**D**) Agarose gel electrophoresis of products from colony PCR amplification of genomic locus containing purA in *T. thermophilus* pTTCC_purA transformants. Expected band size of the KO allele was 1094 bp and the WT allele 2320 bp. (**E**) After growth of pTTCC_purA and pTTCC_crtI transformants in media without antibiotic selection, individual colonies were streaked out on media with and without kanamycin (+ Kan, − Kan, respectively) to reveal the presence or absence of the plasmid. The agarose gel images (**B**,**D**) have been digitally manipulated for clarity. The original images are provided in supplementary file 5.

For generation of a *T. thermophilus crtI* knock-out mutant, a 30 bp spacer complimentary to a sequence in the *crtI* open reading frame was designed and cloned into pTTCC, generating pTTCC_crtI_s. The targeted sequence is followed by 5′-CCTGGAAA consistent with the 5′-NNNNGNMA PAM specificity of CaldoCas9. Subsequently, 0.5 kb sequences flanking the *crtI* open reading frame were amplified and assembled into the plasmid to generate a homologous recombination (HR) construct. The resulting plasmid, pTTCC_crtI, was used for transformation of *T. thermophilus* strain HB27 in three independent experiments. pTTCC (without spacer and HR construct) was used as a control. *T. thermophilus* is naturally competent, and transformation was carried out by mixing the plasmids with the bacterium at 70 °C. After transformation, cells were plated on solid medium with kanamycin and incubated at 65 °C for 24 h for selection of clones carrying the plasmids. For genome editing to occur via the action of CaldoCas9 under these circumstances, the system must therefore be stable in vivo at a minimum of 65 °C. In the three experiments, we obtained 25, 3, and 50 colonies after transformation with pTTCC_crtI and 336, 392 and 166 colonies after transformation with the control plasmid pTTCC, containing neither gRNA spacer or HR construct (average 1490 colonies/µg control plasmid DNA). Induction of Cas9 expression was not performed (the culture medium did not contain cellobiose) and therefore the apparent Cas9 activity was the result of basal level expression from the P_cel_ promoter. Clones that grew on plates after transformation with pTTCC_crtI appeared white, suggesting successful genome editing (Fig. 4A). Since stochastic mutations could possibly occur, e.g., resulting in loss of the megaplasmid with concomitant white phenotype, it was necessary to assess the fidelity and efficiency of potential genome editing in the clones on a molecular level. A PCR based assay was used for this purpose, where the *crtI* gene locus was amplified using primers that bind upstream of the 5′ flanking sequence and downstream of the 3′ flanking sequence used for construction of the HR construct. The primers therefore do not amplify the HR construct in the pTTCC_crtI plasmid. Amplification in the WT locus was expected to yield a 2891 bp amplicon, whereas in the KO locus amplification was expected to yield a 1351 bp amplicon due to deletion of 1540 bp sequence containing the *crtI* open reading frame. Colony PCR was carried out on 22 colonies selected from all three experiments. The smaller amplicon corresponding to the KO allele was observed in 19 cases and no amplification, neither WT or KO allele, was observed from 3 cases (Fig. 4B). Amplification of a different locus on the megaplasmid, approximately 75 kb upstream of *crtI,* and of a chromosomal locus (*purA*), were tested for these three clones (data not shown). No amplification was observed for one clone, suggesting DNA isolation had been unsuccessful in this one instance, and data relating to this clone therefore not informative. Amplification of both loci, on the megaplasmid and on the chromosome, was successful in the other two clones, suggesting that failure to amplify the *crtI* locus in these two instances may result from stochastic deletions on the megaplasmid, encompassing *crtI* and adjacent sequences, but not loss of the megaplasmid altogether. Taken together, these data suggested that in 21 clones, the desired mutation was acquired in 19, whereas two clones contained undesired, stochastic mutations. The observed efficiency of the CaldoCas9 genome editing system in this instance was therefore 90%. The *crtI* locus of two apparent *crtI* KO clones were sequenced. In both cases the desired *crtI* KO mutation was observed (supplementary file 8).

The generation at low frequency of mutants harboring stochastic mutations suggest that DNA repair and survival occurs in a fraction of cells after DNA DSB that is independent of HDR involving the homologous sequences supplied on pTTCC_crtI. Therefore, it would be expected that such stochastic mutants could be generated by transformation of pTTCC_crtI_s (in which CaldoCas9 and *crtI* targeting gRNA are expressed, but no HR sequences are included). In two independent experiments, *T. thermophilus* HB27 was transformed with pTTCC_crtI_s and pTTCC, plated and grown as described previously. After incubation for 48 h, cells were counted. In the two experiments we obtained 9 and 12 colonies after transformation with pTTCC_crtI_s and 115 and 273 colonies after transformation with the control plasmid pTTCC. In total, 18 clones transformed with pTTCC_crtI3 were white and 3 were yellow. The lack of pigmentation suggested genetic mutations had occurred in at least 18 clones. We cultured two white clones transformed with pTTCC_crtI_s in liquid media overnight, isolated DNA and performed PCR assays to investigate the nature of the mutations. Using the primer pair flanking *crtI,* expected to yield a 2891 bp amplicon in WT cells, no amplification was observed (supplementary file 9). The primers bind approximately 0.7 kb upstream and 0.5 kb downstream of the *crtI* start and stop codons, respectively. Therefore, the lack of amplification suggests large-scale genetic deletions had occurred in these mutants. To investigate the extent of the deletion further, we performed PCR with two additional primer pairs. Using a primer pair that amplifies a locus approximately 12 kb upstream of *crtI,* the expected amplicon was observed for one clone and no amplicon for the other. Using a primer pair that amplifies a locus spanning a sequence 0.7–1.7 kb downstream of *crtI,* no amplification was observed for either clone (supplementary file 9). Taken together, these data suggest that following targeted DNA DSB by CaldoCas9 and gRNA in *T. thermophilus*, stochastic mutations may occur, perhaps mediated by HDR with distant sequences in the *T. thermophilus* genome.

We sought to test whether the CaldoCas9 based genome editing system and pTTCC delivery system could be utilized for genome editing on the bacterial chromosome with similar fidelity and efficiency as that observed for editing of the megaplasmid. To that end we aimed to knock out *purA*. We constructed pTTCC_purA containing a gRNA with a 30 bp spacer complimentary to a sequence in *purA* and a HR construct containing 0.5 kb sequences flanking the *purA* open reading frame. In this instance the targeted sequence was followed by 5′-GGAGGACA, again consistent with the PAM specificity of CaldoCas9. *T. thermophilus* was transformed with the plasmid at 70 °C, plated on solid media with kanamycin and grown for 24 h at 65 °C, as before. Again, Cas9 expression was not induced in these experiments. In two separate experiments, 6 and 5 colonies were obtained after transformation of pTTCC_purA. Two colonies were streaked out on minimal media and neither grew without the supplementation of adenine (Fig. 4C), suggesting *purA* knock-out had occurred. Colony PCR was performed on all 11 colonies to assess the fidelity and efficiency of the genetic mutation using primers that bind upstream of the 5′ flanking sequence and downstream of the 3′ flanking sequence used for the HR construct. In total, out of the 11 colonies tested, the PCR based assay suggested that 10 carried the expected KO allele and 1 the WT allele (Fig. 4D). The apparent efficiency of the system in this instance was therefore 91%. The *purA* locus of two clones containing apparent *purA* KO alleles were sequenced. In both cases the desired KO mutation was observed (supplementary file 8).

Taken together, the observed genome editing efficiency was comparable when a gene was deleted on the chromosome (*purA*) and on the megaplasmid (*crtI*). The transformation efficiency was however considerably higher when the CaldoCas9 system was used for deletion on the megaplasmid compared to the chromosome. This may potentially be coincidental, for example related to relative efficiency of the gRNA spacers. Alternatively, it may be associated with the polyploid nature of *T. thermophilus* which possibly results in copy number difference between the megaplasmid and chromosome.

### pTTCC can be used for multiple consecutive genome modifications

For the application of the CaldoCas9 genome editing system for more than a single genome modification, the pTTCC plasmid would need to be introduced and cured in turn to recycle the kanamycin resistance marker. To investigate the feasibility of curing the pTTCC plasmids used for genome editing (pTTCC_crtI and pTTCC_purA), their stability in cells grown without selection pressure was assessed. Two clones, one *crtI* KO and one *purA* KO, were grown separately in liquid media without antibiotic selection overnight at 70 °C. It was assumed that in the absence of antibiotic selection pressure, plasmid curing would occur, at least in a considerable proportion of the cells. They were diluted the next day and plated on solid media without antibiotic and grown for 24 h at 70 °C. Individual colonies were assayed for the presence of the pTTCC_crtI and pTTCC_purA plasmids, by re-streaking on plates with and without kanamycin. Clones retaining the plasmids would be expected to grow on both plates, whereas in clones where plasmid curing had occurred no growth would be expected on plates with kanamycin. For *crtI* KO clones, we observed apparent plasmid curing in all 4 colonies tested, and for *purA* KO clones, we observed plasmid curing in half (2/4) of the colonies tested (Fig. 4E). This suggests that in the absence of selective pressure, pTTCC is not stable and is cured in a majority of cells after an overnight growth in liquid media. As a result, multiple consecutive rounds of genome editing can be achieved with the thermostable CRISPR-Cas9 system carried on pTTCC.

## Discussion

Thermophiles are studied for elucidation of the molecular mechanisms underlying life at elevated temperatures; for exploration of limits of life, e.g., in the context of life on other planets and the origin of life; and for their potential in the field of industrial microbiology, as whole cell catalysts for conversion of feedstocks to diverse, useful and valuable chemicals. Genetic tools enabling insertion, deletion and substitution of genetic material are necessary to achieve the full potential of these research fields. However, tools for genome editing of thermophilic organisms, where available, are largely immature^28^. At elevated temperatures, as well as at high salt concentrations, extremes of pH, or absence of oxygen, frequently required for growth of thermophiles, many of the molecular components of genetic tools used in mesophilic species lose their activity. This includes some antibiotics, antibiotic resistance enzymes, and fluorescent proteins. As a result, development of genetic tools for thermophiles is limited to a smaller number of molecular components compared to mesophiles.

In the past decade considerable progress has nevertheless been made in the development of genome editing tools for thermophiles. Tools are available that allow multiple consecutive rounds of genome editing in thermophiles of genera *Thermus*, *Sulfolobus, Thermococcus* and *Pyrococcus*, and progress towards this end has been reported for thermophiles of genera *Rhodothermus, Thermoanaerobacter, Caldicellulosiruptor,* and *Thermotoga*^28^. The majority of these systems rely on selection of antibiotic resistant/sensitive strains using thermostable antibiotics (kanamycin, hygromycin, bleomycin, and simvastatin) and thermo-adapted variants of their respective resistance enzymes and/or on selection of nutrient auxotrophic/heterotrophic strains (uracil, tryptophan, agmatine) using minimal media, the respective nutrient, toxic nutrient derivatives (5-fluoroorotic acid, 6-Methyl purine), and metabolic enzymes^28^. Where possible, recycling of selectable markers for a consecutive round of genome editing is however generally time consuming as it can involve screening of many clones for the desired mutation, repeated re-streaking to obtain pure clones, and/or it may require specific genetic mutant background strains, or specialized media components.

The aerobic thermophilic species belonging to the genus *Thermus* grow at circumneutral to alkaline pH, at temperatures ranging approximately from 60 to 85 °C. This is one of the most widespread genera of thermophilic bacteria and is found in natural geothermal areas as well as in man-made thermal environments^44^. The genus is a rich source of catabolic genes and gene clusters encoding enzymes for utilization, of diverse carbohydrates and their cellular import. It also has important precursor pools for terpenoid and carotenoid synthesis^45^, hydroxyalkonates, rhamnolipids^46^ and compatible solutes^47^. The high growth rates and cell yields of the *Thermus* spp. cultures, the small genome size, and the constitutive expression of an impressively efficient natural competence apparatus, make strains of the genus excellent laboratory models to study the molecular basis of life at elevated temperatures, genetic manipulation, structural genomics, systems biology and synthetic biorefinery potential^48^. *T. thermophilus* strains contain a number of polymer degrading enzymes, α-amylases, xylanases, esterases, lipases, proteases and pullulanases and are therefore able to grow on different organic sources such as various proteinaceous and carbohydrates substrates. The genetic tractability and the number of genetic tools that have already developed for strain HB27 makes it an ideal thermophilic chassis species to study and subsequently develop for biorefinery usage using tools of system and synthetic biology.

Many genome editing systems that use selection-counterselection strategies have been developed for *T. thermophilus:* in a Δ*pyrE* strain, a gene targeted for deletion was replaced by *pyrE* and uracil prototrophs selected in minimal medium, followed by counterselection to remove *pyrE* using 5-fluoroorotic acid^49^; A construct containing *rpsL1*a, a dominant allele which confers a streptomycin (Str) dependence phenotype, was integrated into a target locus and Str resistant clones selected, followed by counterselection in Str-free media to remove the *rpsL1* construct along with the target gene^50^; In a Δ*bgl* strain, a construct containing *bgl* and a kanamycin resistance allele was integrated into a target locus and kanamycin resistant clones selected, followed by counterselection using 5-bromo-4-chloro-3-indolyl-β-glucoside to remove the integrated construct and target gene^51^; A construct containing *pheS2* (which causes sensitivity to a phenylalanine analog) and a kanamycin resistance allele was used to replace a target gene and kanamycin resistant clones selected, followed by counterselection to remove the construct using *p*-chlorophenylalanine^52^; A construct containing *codA* (which causes sensitivity to 5-fluorocytocine) and a kanamycin resistance gene was integrated into a target locus and kanamycin resistant clones selected, followed by counterselection using 5-fluorocytocine to remove the construct and target gene^43^. Other genome editing systems that have been developed for *T. thermophilus* and involve recycling of selection markers do not rely on counterselection: In strains carrying mutations in the carotenoid biosynthesis pathway, white and orange colony color was used in turn to identify edited clones^53^; genes were deleted using a Cre/*lox* based site-specific recombination^54^; and genes were deleted using double crossover homologous recombination constructs without selection markers, and edited clones identified via mass screening PCR^55^. Taken together, there exist a wealth of diverse methods that enable metabolic engineering of *T. thermophilus.* Their use however presents some challenges that can be time consuming to address. In particular: some of these methods require specific genetic mutant strains^49,51–53^; spontaneous mutants can arise that are resistant to counter-selection or harbor otherwise unwanted mutations^50,52,54^; where necessary, growth in minimal media or specialized growth conditions requires cultivation for several days^43,54^; where a single ‘pop-in / pop-out’ genetic recombination construct is used, theoretical yield of mutants cannot surpass 50%^43,50,51^ and some methods require design and construction of two separate genetic recombination constructs^49,52,53^.

In contrast, the thermostable CRISPR-Cas9 based genome editing system presented herein can be used in WT or mutant strains, transformants are grown rapidly in rich media, pTTCC enables simple construction and delivery of the genome editing system, and the apparent yield of desired mutations is very high. We nevertheless observed a low frequency of unwanted mutations and apparent background of WT cells. Using more stringent selection (higher kanamycin concentration) may potentially reduce the growth of WT background colonies, but in our hands this comes at the cost of considerably fewer transformants (data not shown). We hypothesize that the observed undesired mutations arose following Cas9 induced double strand break and is the result of an inaccurate DNA repair mechanism, perhaps utilizing homologous recombination of somewhat divergent genomic sequences.

Various conditions relating to the use of pTTCC for genome editing of *T. thermophilus* remain to be tested that could potentially affect the genome editing efficiency, transformation efficiency, or both. These conditions include induction of CaldoCas9 and varying gRNA spacer length. pTTCC includes an inducible promoter regulating the transcription of CaldoCas9. In our experiments using pTTCC we did not test induction of CaldoCas9, since basal level expression proved sufficient for the function of the system. We reasoned that increasing CaldoCas9 activity by induction would potentially increase the risk of off-target effects, which would decrease transformation efficiency or increase the incidence of unwanted off-target mutations. Increasing the activity of CaldoCas9 by induction could however increase genome editing efficiency since it would increase mortality among non-recombined cells. In our experiments, genome editing efficiency was however very high, and it is therefore not obvious that induction is beneficial. In our experiments we used CaldoCas9 in combination with a gRNA containing an unconventionally long spacer, 30 bp. RNA sequencing data from *G. stearothermophilus* suggests spacer length of crRNAs in the bacterium is most commonly 22–24 bp and the in vitro activity of GeoCas9 is highest in combination with gRNAs with 21–22 bp spacers^19^. Using shorter gRNA spacers with CaldoCas9 may therefore possibly increase the enzyme’s activity. In relation to use of the system for genome editing, this could result in increased genome editing efficiency. Again, the observed high efficiency in our experiments suggest that CaldoCas9 with gRNAs containing 30 bp spacers is however sufficiently active to eliminate unedited clones, and it may therefore be unnecessary to increase its activity in this context.

The use of CRISPR-Cas9 systems for efficient genome editing of extreme thermophiles at temperatures above 55 °C has to our knowledge not been reported previously. In vivo activity of GeoCas9 and ThermoCas9 has been reported at a maximum temperature of 50–55 °C^18,23^, and in an extreme thermophile CRISPR-Cas9 based genome editing was inefficient^24^. We showed that application of the CaldoCas9 genome editing system is possible in organisms grown at temperatures of at least 65 °C. The CaldoCas9 genome editing system could in theory be adapted for use in any organism, given that the enzyme and associated gRNA can be delivered or expressed in the cells, and that they are active at the organism’s growth temperature. The CaldoCas9 genome editing system may therefore facilitate genome editing of other thermophilic organisms, including those for which limited tools exist to date. Adaptation of the system for plasmid-based delivery in other organisms would require availability of an appropriate replication plasmid, a positive selection marker and promoters for regulation of CaldoCas9, gRNA and selection marker expression. The applicability of CaldoCas9 based genome editing in different species will in part depend on the activity of endogenous repair mechanisms, like other CRISPR-Cas genome editing systems. It may therefore in some instances be facilitated by co-expression of recombinases^16^. CaldoCas9, like other Cas9 enzymes is a large polypeptide, which could potentially be difficult to express in some cells. CaldoCas9 and other Cas9 enzymes have however been successfully expressed in diverse bacterial species^56^ and other organisms. Adaptation of the CaldoCas9 genome editing system in other thermophilic bacteria that are closely related to *T. themophilus* may be relatively simple, involving limited or no modifications where activity/sequence of promoters, origins of replication, etc., are well conserved. In this context, it is notable that *T. thermophilus* strain HB8 is widely studied, similar to HB27. We hypothesize that the CaldoCas9 genome editing system and pTTCC plasmid can be used without modification in other *T. thermohilus* strains, including HB8, but this remains to be investigated.

A large proportion of prokaryotic species express endogenous CRISPR-Cas systems, in particular of types I and III^6,57,58^. Endogenous CRISPR-Cas systems have been exploited for genome editing in bacteria and archaea, including in extreme thermophiles^59–63^. Development of such systems may further facilitate genome engineering of thermophiles, and could be used in conjunction with Cas9 genome editing systems such as Caldocas9 to increase the number of targetable genomic loci beyond those containing the respective Cas9 PAM. The *T. thermophilus* HB27 genome encodes both type I and type III CRISPR-Cas systems^37^. An alternative genome editing system to that presented herein could therefore potentially be designed for *T. thermophilus* that utilizes these endogenous systems. As endogenous CRISPR based genome editing systems do not require exogenous expression of a nuclease they can be delivered on much smaller plasmids (or other delivery vectors). This may have benefits over the CaldoCas9 based system in that it could facilitate delivery of larger homologous recombination constructs, thus enabling larger genomic insertions.

Thermostable Cas9 enzymes may have applications other than for genome editing of thermophilic organisms. Their stability in human plasma, and potential therapeutic use in humans has been noted previously^19^. Cas nucleases are used for a wide range of applications, including in diagnostic tests^64^. Thermostability to increase shelf life of Cas9 based detection kits may be desirable in some instances. Finally, to achieve the full potential of Cas9 applications, it is important to further increase targetable nucleic acid sequences. In that context, previously identified Cas9 enzymes can be engineered or new enzymes identified that carry novel PAM specificities. The PAM specificity of CaldoCas9, 5′-NNNNGNMA, has not been described in other Cas9 enzymes to our knowledge.

## Materials and methods

### Bacterial strains, culture conditions and transformation

*E. coli,* NEB Stable (NEB C3040) and NEB 5-alpha (NEB C2987), were grown in L media at 37 °C, with 30 µg/ml kanamycin when appropriate. Transformation of *E. coli* was performed according to manufacturer’s instructions. *T. thermophilus* strain HB27 was grown in *Thermus* broth (TB): 8 g/L tryptone (Accumedia MC005), 4 g/L yeast extract (Bacto, 212750), 3 g/L NaCl (Fluca, 31,434), pH 7.5, with 23 µg/ml kanamycin when appropriate. Transformation of *T. thermophilus* was performed as described previously^35,65,66^: a single colony of *T. thermophilus* from a fresh culture plate was suspended in 10 ml TB and grown overnight at 70 °C with shaking. The following day, the culture was diluted by mixing 200 µl of the overnight culture in 10 ml fresh TB. The diluted culture was incubated at 70 °C with shaking until optical density at 550 nm reached 0.8 (approximately 4 h). A 500 µl aliquot of the culture was mixed with 200 ng of plasmid and culture continued at 70 °C with shaking for 2 h. The cells were plated on TB agar plates with kanamycin and incubated at 65 °C for 16–24 h.

### CaldoCas9 expression and purification

The *Geobacillus* sp. strain LC300 *cas9* gene (genome and protein reference numbers: NZ_CP008903, WP_050368141) was ordered synthesized, codon optimized for high %GC (supplementary file 7). It was synthesized by Eurofins genomics and supplied on a replication plasmid pEX-K4. The gene was cloned into an in-house plasmid, pHWG1106, under the control of a rhamnose inducible promoter to generate pHWG1106_MBP-SMT3-CaldoCas9 (supplementary file 10). It was cloned in frame with additional sequences such that CaldoCas9 was expressed as a fusion protein MBP-SMT3-CaldoCas9 (N- to C- terminus), where MBP is maltose/maltodextrin-binding periplasmic protein and SMT3 is Ubiquitin-like protein SMT3. The fusion protein was 1561 residues and 179.2 kDa. MBP facilitates protein folding and affinity purification; SMT3 enables removal of the MBP fusion via Ulp1 protease cleavage^67^. Cloning of the CaldoCas9 gene into pHWG1106 was performed by Gibson assembly. The gene was amplified from pEX-K4 with primers F_GA_gbCas9Ropt_malE_pHWH1106 and R_GA_gbCas9Ropt_malE_pHWH1106 (supplementary file 11) with a high-fidelity polymerase, and the amplicon assembled into *Bam*HI, *Sfo*I (NEB, R0136 and R0606) digested pHWG1106 vector, according to manufacturer’s instructions (NEB, E2611). The pHWG1106_MBP-SMT3-CaldoCas9 plasmid was introduced into *E. coli* strain BL21 C43 by transformation, gene expression induced with rhamnose and cells harvested. Cells were sonicated in a mild buffer (20 mM HEPES, 10 mM NaCl, 0.5 mM MgCl_2_, 0.01 mM EDTA, pH 6.5, 5 ml per gram cell pellet) and spun to remove cell debris. The supernatant was purified on an amylose column under control of Äkta purifier with UNICORN software: with sample binding in 20 mM Tris, 200 mM NaCl, pH 7.6 buffer; elution with a linear gradient 0–100% at 0.8 ml/min of elution buffer composed of 20 mM Tris, 200 mM NaCl and 10 mM maltose, pH 7.6. The purity of the eluted protein was assessed with SDS-PAGE and eluted fraction EF4 was used for in vitro experiments (supplementary file 12).

### gRNA transcription and purification

The gRNA for CaldoCas9 was synthesized by PCR amplification using a scaffold DNA and two primers. A 194 bp gRNA scaffold was ordered synthesized, containing the whole gRNA sequence excluding the spacer (supplementary file 13). A forward ‘spacer primer’ was designed that contained a T7 RNA polymerase promoter, a gRNA spacer sequence, and a part of the gRNA stem and was thus partially complimentary to the scaffold. A PCR using the ‘spacer primer’ and a reverse primer (R_sgRNA_scaffold, supplementary file 11) and the gRNA scaffold as template generated the full-size gRNA DNA amplicon. The spacer sequence of the ‘spacer primer’ could be changed to generate gRNAs targeting any desired sequence. gRNAs were synthesized for in vitro cleavage assays using primers MM_gbsgRNA_rtTA_2 and crtI_TT_s3_F (non-target gRNA), and for PAM library targeting using primer GBsgRNAs6 (supplementary file 11). RNA was generated by in vitro transcription of the amplicons using T7 polymerase and purified as described in the Guide-it IVT RNA Clean-Up Kit (Takara, 632638).

### In vitro DNA cleavage assays

In vitro DNA cleavage assays were performed in a Cas9 reaction buffer (20 mM HEPES, 10 mM NaCl, 0.5 mM MgCl2, 0.01 mM EDTA, pH 6.5). The reaction mixture contained 1 µl gRNA (50 ng/µl), 1 µl CaldoCas9 EF4, 1.5 µl Cas9 reaction buffer (10×), 100 ng target DNA and water to 15 µl final volume. The reactions were incubated for 1 h at a desired temperature. Before removal from the heating-blocks, gel loading dye containing SDS and EDTA (NEB, B7024) was added to quench the reactions. The reaction products were separated on agarose gels, visualized with SYBR safe (ThermoFisher, S33102), using a DNA ladder for reference (NEB N3232), and imaged on a UV dock (BIO-RAD GelDoc XR +). The original images (supplementary file 5) were processed for enhanced contrast and clarity.

### Determination of Cas9 PAM specificity

Determination of CaldoCas9 PAM specificity was conducted as described by Karvelis et al.^32^. The methodology involves creating plasmid libraries containing a target sequence for Cas9 mediated DNA double strand break and a randomized PAM sequence; digestion of the libraries with Cas9; isolation and amplification of the linearized DNA strands; and sequencing. As only linearized fragments are sequenced, their sequences reveal the PAM specificity of Cas9.

Three oligonucleotides were constructed, gbS6_PAMseq_pos1to4_R, gbS6_PAMseq_pos5to8_R, gbS6_PAMseq_pos8to12_R (supplementary file 11) each containing the same target sequence followed by randomized downstream nucleotides in positions 1–4, 5–8 or 9–12 (Fig. 2C). To generate a double stranded DNA fragment containing the target and randomized PAM, the three oligonucleotides were mixed with the partially complimentary gbS6_PAMseq_F (supplementary file 11) and non-complimentary sequences filled-in with Taq polymerase (NEB M0273). The reaction was composed of 0.2 µM each oligonucleotide, 0.6 U Taq polymerase, and 0.2 mM dNTPs in a total volume of 25 µl with 1 × standard Taq reaction buffer. It was heated to 95 °C for 30 s, cycled 20 × (at 95 °C, 52 °C and 68 °C for 30 s each), and finally extended at 68 °C for 20 min. Amplicons and vector pTZ57R/T (CloneJet PCR cloning kit, ThermoFischer, K1231) were digested with *Bam*HI and purified and the amplicons ligated into the vector using T4 DNA ligase (NEB M0202) at a molar ratio of 3:1 (approximately 0.06 pmol vector and 0.2 pmol insert), respectively. *E. coli* NEB Stable competent cells (NEB C3040) were transformed with 5 µl of the ligation product, plated on L-media with agar and ampicillin, 100 µg/ml, and incubated overnight at 37 °C. The libraries contained four randomized bases each, and therefore consisted of 256 different molecules. We aimed to obtain at least 2500 transformant colonies to ensure a theoretical 10 × representation of each molecule. For each of the three libraries transformed, we obtained 3000–4000 colonies. They were resuspended in 3 ml L media and miniprepped, and referred to as libraries N1-4, N5-8 and N9-12 (Fig. 2C).

The miniprepped libraries were digested by CaldoCas9. For each of the three libraries, two mixtures were prepared: one containing 2 µl of CaldoCas9 EF4 and 1 µl gRNA (50 ng/µl), and a second containing 500 ng library DNA, 1 × Cas9 reaction buffer and water to a final volume of 12 µl. The two mixtures were incubated separately for 5 min before they were combined and incubated for 4 h, both at 57 °C. Finally, the reactions were quenched by heating to 80 °C for 5 min. The linearized (and thus PAM containing) DNA molecules were captured from the reactions by A-tailing with Taq, followed by adapter ligation. A-tailing was performed by incubating the 15 µl Cas9 reactions with 2.5U Taq polymerase and 0.5 µl 10 mM dATP for 30 min at 72 °C. Adapters were generated by annealing of two oligonucleotides, TK-117 and TK-111 (supplementary file 11). They were then ligated to the A-tailed linearized libraries with T4 ligase using 100 ng of each reactant. The linearized, A-tailed and adapter ligated libraries were enriched by PCR amplification using Q5 polymerase (NEB, M0491) and primers pUC-dir and TK-117 (supplementary file 11). The amplicons were purified, MiSeq barcodes added by Q5 amplification with primers pUCdir-seq and TK-117-seq (supplementary file 11) and sequenced on Illumina MiSeq instrument. The resulting reads were analyzed with Geneious 9.1.8 software to reveal the PAM specificity of CaldoCas9. Reads containing 100% identity across nucleotides comprising the target sequence and 12 downstream bases were ‘filtered’ from the remaining data and analyzed. They were 389 for library N1–4, 510 for library N5–8 and 645 for library N9–12. The coverage per base was therefore around 100-fold or over for all libraries. To measure frequency of each nucleotide in the 3′-sequence of libraries prior to Cas9 digestion, they were PCR amplified with primers pUC-dir and R_gblib_PAMverif (supplementary file 11; indicated as P1 and P2 in Fig. 2C) and subsequently A-tailed, adapter ligated and sequenced as described for the Cas9 digested libraries. The frequency of each base in the digested library was normalized to the frequency in the undigested library to correct for bias in the latter (supplementary file 6). The normalized frequency was used to generate graphical representations of the PAM preference using WebLogo^68^.

### Construction of pTTCC

pTTCC was constructed using an *E. coli, T. thermophilus* shuttle-expression vector, pLEI250.2^69^, as a backbone (5371 bp). The vector backbone contained: origins of replication for maintenance (ORI) in *E. coli* and *T. thermophilus*; a kanamycin resistance gene; and *bglT* under regulation of a cellobiose inducible promoter and a transcription terminator sequence. *bglT* was flanked by *Hind*III and *Nde*I restriction sites. We removed a *Bbs*I restriction site located in the *T. thermophilus* ORI in pLEI250.2 using site-directed mutagenesis as described in^70^ with primers F_pLEI250delBbsI and R_pLEI250delBbsI (supplementary file 11) and high-fidelity polymerase to create pLEI250.2-BbsI. The primers contained a single nucleotide mismatch with the template, located in the *Bbs*I recognition site. Methylated template DNA was removed after the reaction by restriction using *Dpn*I (R0176, NEB) and NEB5-alpha competent *E. coli* (C2987, NEB) transformed with the amplicons. Transformants were grown in liquid media, plasmids miniprepped, and removal of the restriction site confirmed with *Bbs*I digestion. The gene encoding CaldoCas9 was amplified from pEX-K2 with primers F_gbCas9R_NdeI and R_gbCas9R_HindIII with Q5 polymerase and ligated into *Nde*I and *Hind*III restricted pLEI250.2-BbsI with T4 ligase, to create pLEI251. NEB Stable competent *E. coli* were transformed with the ligation mixture. Transformants carrying the desired insert were identified with colony PCR. A *Bbs*I restriction site in the gene expressing CaldoCas9 was removed on pLEI251 to create pLEI252, using Q5 site-directed mutagenesis (NEB, E0554) with primers Q5SDM_4/13_F and Q5SDM_4/13_R following manufacturer’s instructions. Transformants were grown in liquid media, plasmids miniprepped and elimination of the restriction site confirmed by *Bbs*I digestion. To create pTTCC, vector pLEI252 was digested with *Kpn*I and a 477 bp DNA fragment (synthesized by Eurofins) inserted with HiFi assembly (E2621, NEB) according to manufacturer’s instructions and transformed in *E. coli* NEB Stable cells. The DNA fragment (supplementary file 14) contained a construct for gRNA spacer cloning and expression under regulation of P_trc_. Two *Bbs*I restriction sites flank a gene encoding a lacZα fragment, an approach similar to (Altenbuchner 2016)^71^. Transformants carrying pTTCC were identified with colony PCR (pLEIseqR_trcgRNA and R_sgRNA_scaffold, supplementary file 11) and cultured in liquid media overnight. Plasmids were miniprepped and sequenced across the inserted fragment (pLEIseqR_trcgRNA, supplementary file 11).

### Genome editing of Thermus thermophilus with pTTCC

Spacers were cloned into pTTCC using restriction ligation of oligonucleotides into restriction digested pTTCC (details in supplementary file 15). Briefly, oligonucleotides were designed containing the desired spacer sequence. They were phosphorylated and annealed to generate an oligo duplex. pTTCC was digested with *Bbs*I-HF (NEB, R3539) and the duplex ligated into the vector with T4 DNA ligase. NEB Stable cells were transformed with the ligation mixture according to manufacturer’s instructions (C3040) and plated on L agar media with 30 µg/ml kanamycin and cultured overnight at 37 °C. A few clones were cultured in liquid media overnight at 37 °C and miniprepped (NEB T1010). Insertion of the desired spacer sequence was confirmed by Sanger sequencing using pLEIseqR-trcgRNA. In this manner, oligonucleotides crtI_TT_s3_F and crtI_TT_s3_R2 (supplementary file 11) were inserted into *Bbs*I restricted pTTCC to generate pTTCC_crtI_s; and to generate pTTCC_purA_s the oligonucleotides were PurA_Spacer_2_F and PurA_Spacer_2_R (supplementary file 11).

A construct for directing homologous recombination resulting in gene knock-out was composed of two approximately 500 bp DNA sequences homologous to genomic regions flanking the target genes. The two sequences were amplified separately with Q5 DNA polymerase according to manufacturer’s instructions, using *T. thermophilus* strain HB27 genomic DNA as template. Genomic DNA was isolated from an overnight liquid culture of strain HB27 using Masterpure Complete DNA and RNA Purification kit (MC85200, Epicentre). For amplification of sequences flanking *crtI*, primers F2_GA_crtI3end, R2_GA_crtI3end, F2_GA_crtI5end and F2_GA_crtI5end were used, and for amplification of sequences flanking *purA*, primers were PurA_5F, PurA_5R, PurA_3F, PurA_3R (supplementary file 11). Plasmids pTTCC_crtI_s and pTTCC_purA_s were restricted with *Kpn*I-HF (NEB, R3142) according to manufacturer’s instructions. The restricted vector and amplicons were each separated from their respective reagent mixtures by agarose gel electrophoresis; cut from the gels and purified using Monarch DNA Gel Extraction Kit (T1020, NEB). The purified amplicons and vector were assembled (HiFi, NEB) using two-fold molar excess of amplicons to generate pTTCC_crtI and pTTCC_purA. NEB 5-alpha Competent *E. coli* (NEB, C2987) were transformed with the assembly mixture, plated on L media with agar and kanamycin (30 µg/ml) and incubated at 37 °C overnight. To identify clones containing the insert, colony PCR was performed with primers pTTCC_HR_ampF and pTTCC_HR_ampR. A few clones were cultured overnight in liquid L media with kanamycin (30 µg/ml) and plasmids miniprepped (NEB, T1010). The miniprepped plasmids were restricted with *Bsp*HI (NEB, R0517) and separated on agarose gel to confirm presence of inserts.

Plasmids pTTCC_purA and pTTCC_crtI were used to generate *T. thermophilus* KO mutants. To confirm successful genome editing, a colony PCR based assay was used. To isolate DNA, *T. thermophilus* transformant colonies were: picked into 20 µl 10% chelex solution (BT Chelex 100, Bio-Rad 143-2832) and suspended by vortexing; incubated at 55 °C for 15 min; vortexed again and incubated at 100 °C for 10 min; placed on ice for 3 min; spun at 11,000×*g* for 7 min and the supernatant containing DNA collected. PCR was performed using the isolated DNA to determine allele size in the *crtI* and *purA* genome loci. For putative *crtI* KO mutants primers F_Tthp_60 and R_Tthp_60 were used and for putative *purA* KO mutants primers PurA_Verify_F and PurA_Verify_R were used (supplement file 11). To confirm the presence of the megaplasmid in mutants where amplification of the *crtI* locus was not observed, primers bgal_amp_F and bgal_amp_R were used. The reaction products were separated on agarose gels, visualized with SYBR safe (ThermoFisher, S33102), using a DNA ladder for reference (NEB N3232), and imaged on a UV dock (BIO-RAD GelDoc XR +). The original images (supplementary file 5) were processed for enhanced contrast and clarity.

### Culture in minimal media and plasmid curing

To determine whether *purA* knock-out was successful, resulting in adenine auxotrophy, *T. thermophilus* was cultured in minimal medium with and without adenine supplementation. Colonies were streaked on agar plates containing 10% medium 162^72^ with modifications (2 mM MgSO_4_ and 0.2 mM CaCl_2_ in final volume), with addition of 0.3% NaCl, 8 mM phosphate buffer (KH_2_PO_4_, Na_2_HPO_4_, pH = 7.2), 10 mM NH_4_Cl, 0.2% casamino acids, 0.4% glucose and 0.001% Wolfe´s vitamin solution^73^. Agar plates were supplemented with 100 µl of 0.5% adenine solution before spreading cells, when applicable.

To cure plasmids pTTCC_crtI and pTTCC_purA from transformants, colonies were placed in 10 ml TB media and cultured overnight at 70 °C and shaking at 200 rpm. The cultures were diluted 100- and 1000-fold in fresh TB medium, 50 µl plated on TB agar plates and cultured for 24 h at 70 °C. To test whether plasmid curing was successful, single colonies were finally streaked on TB agar media with and without supplementation of 23 µg/ml kanamycin and cultured for 24 h at 70 °C.

### Digital image processing

Figures were compiled using GIMP 2.8.22 (gimp.org). Drawn schematics (Figs. 1F, 2C, supplementary file 1) were created by author BTA using GIMP. Agarose gels were processed using ImageJ 1.51j8 (imagej.nih.gov/ij). Schematics in Fig. 1A,C–E were created in Geneious 9.1.8 (geneious.com). Plasmid maps (Fig. 3) were created using Snapgene 5.0 (snapgene.com). A sequence logo (Fig. 2C) was created with weblogo 2.8.2 (weblogo.berkeley.edu).

## Supplementary Information

Supplementary Information

## Data Availability

The datasets generated during and/or analysed during the current study are available from the corresponding author on reasonable request.
