# Supplementary material for: Efficient genome editing of an extreme thermophile, Thermus thermophilus, using a thermostable Cas9 variant
Source: Sci Rep. 2021 May 5;11:9586. doi: 10.1038/s41598-021-89029-2 (PMC8100143; doi:10.1038/s41598-021-89029-2)
Supplement: Supplementary file 1 — Supplementary Information [file 41598_2021_89029_MOESM1_ESM.pdf]

Supplementary files from manuscript:
Efficient genome editing of an extreme thermophile, *Thermus thermophilus,* using a thermostable Cas9 variant

Bjorn Thor Adalsteinsson[1]*, Thordis Kristjansdottir[1,2], William Merre[3], Alexandra Helleux[4], Julia Dusaucy[5], Mathilde Tourigny[4], Olafur Fridjonsson[1], Gudmundur Oli Hreggvidsson[1,2]
1. Matís, Reykjavík, Iceland; 2. University of Iceland, Reykjavík, Iceland; 3. Aix-Marseille Université, Marseille, France; 4. Université de Strasbourg, Strasbourg, France; 5. Université Pierre et Marie Curie, Paris, France; *corresponding author (bjornth@matis.is)

Supplementary file 1

A generalized scheme of Cas9 genome editing in prokaryotes, using a plasmid based delivery system with kanamycin selection:

I. Transformation, expression, antibiotic selection

After transformation with a Cas9 genome editing plasmid, Cas9, gRNA and an antibiotic resistance gene (kanamycin in schematic) are expressed, and the plasmid is replicated.

Cells carrying the plasmid are selected by plating on media containing antibiotic. Cells that do not carry the plasmid die.

II. Cas9 DSB and HDR

Cas9 and gRNA cause a double strand break (DSB) in the targeted genomic locus. Three outcomes from this event are possible:

- Homology directed repair (HDR) occurs between the genome and a construct supplied on the plasmid (this scenario is demonstrated in schematic). This leads to a 'controlled' genome editing.
- Repair occurs independent of the construct supplied on the plasmid. Genome editing is not 'controlled'. Depending on genomic context, the cell may or may not survive (this scenario is not demonstrated in schematic).
- No repair occurs and the cell dies (this scenario is not demonstrated in schematic).

III. Outcome

In the example demonstrated the Cas9 genome editing plasmid was designed to generate a gene knock-out (KO). In the mutants, expression continues from the replicated plasmid, enabling continued growth on media containing antibiotic.

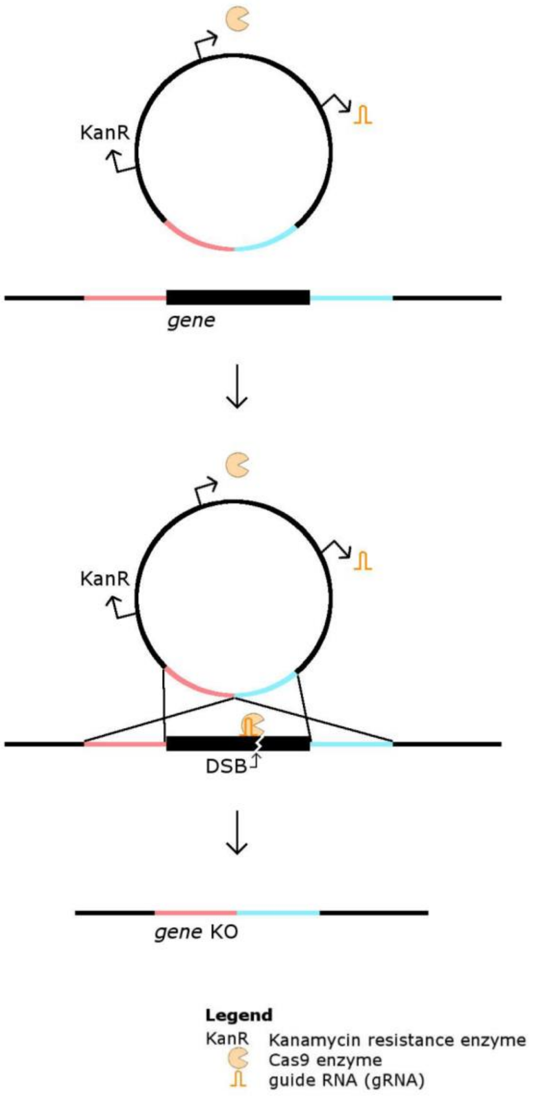

Notes:

If Cas9 and/or gRNA were absent in the scenario depicted above, HDR may occur regardless in a small subset of cells. However, without the negative selection of Cas9:gRNA, both mutants and WT clones would grow. Unless the genome editing in question elicits phenotypic change, mutants must be distinguished from WT clones via genetic screening.

If a homologous recombination construct was absent in the scenario depicted above, the DSB would be fatal to cells unless: they perform non-homologous end-joining repair (which is rare in prokaryotes) leading to an indel mutation; or they perform HDR between the targeted locus and another locus in the genome, leading to stochastic and potentially large-scale genomic alterations.

Supplementary file 2

cas9 genes in *Geobacillus* species

Accession	% Identical Sites	Organism	strain
NZ_CP014335	87.90%	*Geobacillus thermoleovorans*	KCTC 3570
NZ_JPYA01000050	86.80%	*Geobacillus icigianus*	G1w1
NZ_LQYX01000092	86.80%	*Geobacillus sp.*	B4113_201601
NZ_JQMN01000001	86.80%	*Geobacillus subterraneus*	PSS2
NZ_JYBP01000003	87.10%	*Geobacillus kaustophilus*	Et7/4
NZ_JYCF01000004	87.50%	*Geobacillus kaustophilus*	Et2/3
NZ_BCQG01000002	87.60%	*Geobacillus jurassicus*	NBRC 107829
NZ_AYSF01000013	88.40%	*Geobacillus sp.*	MAS1
NZ_LQIE01000074	88.70%	*Geobacillus stearothermophilus*	C1BS50MT1
NZ_JYNW01000007	88.90%	*Geobacillus stearothermophilus*	ATCC 12980
NZ_LLKS01000054	88.90%	*Geobacillus sp.*	Sah69
NZ_JALS01000081	88.90%	*Geobacillus stearothermophilus*	ATCC 7953
NZ_LQYV01000072	89.00%	*Geobacillus stearothermophilus*	B4109
NZ_LUCR01000150	89.00%	*Geobacillus stearothermophilus*	GS27
NZ_CP008903	89.00%	*Geobacillus sp.*	LC300
NC_022080	100.00%	*Geobacillus sp.*	JF8

Query coverage was 100%, except for sequence in genome NZ_CP014335 where it was 86%.

Supplementary file 3

Cas9 enzymes in *Geobacillus* species

**Accession**	**% Identical Sites**	**Organism**	**Strain**
AKU27017.1	100%	*Geobacillus sp.*	LC300
WP_100664518.1	99%	*Geobacillus sp.*	WSUCF-018B
WP_138187407.1	99%	*Geobacillus thermoleovorans*	RL
WP_064213580.1	98%	*Geobacillus stearothermophilus*	GS27
WP_033016936.1	98%	*Geobacillus stearothermophilus*	ATCC 7953
WP_055358891.1	98%	*Geobacillus sp.*	Sah69
WP_121625896.1	98%	*Geobacillus stearothermophilus*	FHS-PCGT429
WP_096225890.1	98%	*Geobacillus sp.*	FJAT-46040
WP_053532223.1	98%	*Geobacillus stearothermophilus*	ATCC 12980
KZE96909.1	97%	*Geobacillus stearothermophilus*	C1BS50MT1
WP_095858800.1	97%	*Geobacillus stearothermophilus*	DSM 458
WP_023633350.1	96%	*Geobacillus sp.*	MAS1
WP_144329852.1	96%	*Geobacillus sp.*	LEMMJ02
WP_066227285.1	95%	*Geobacillus jurassicus*	NBRC 107829
WP_044736072.1	95%	*Geobacillus kaustophilus*	Et2/3
WP_041267823.1	94%	*Geobacillus sp.*	JF8
AGT30711.1	94%	*Geobacillus sp.*	JF8
WP_081209836.1	94%	*Geobacillus sp.*	46C-Iia
WP_033844707.1	93%	*Geobacillus subterraneus*	PSS2
WP_033018780.1	93%	*Geobacillus icigianus*	G1w1
WP_081157433.1	89%	*Geobacillus sp.*	47C-Iib
WP_087959824.1	89%	*Geobacillus thermodenitrificans*	T12
WP_099233044.1	88%	*Geobacillus thermodenitrificans*	ID-1
KJE28979.1	94%	*Geobacillus kaustophilus*	Et7/4

Query coverage was 100%, except for sequence KJE28979.1 where it was 79%.

Supplementary file 4

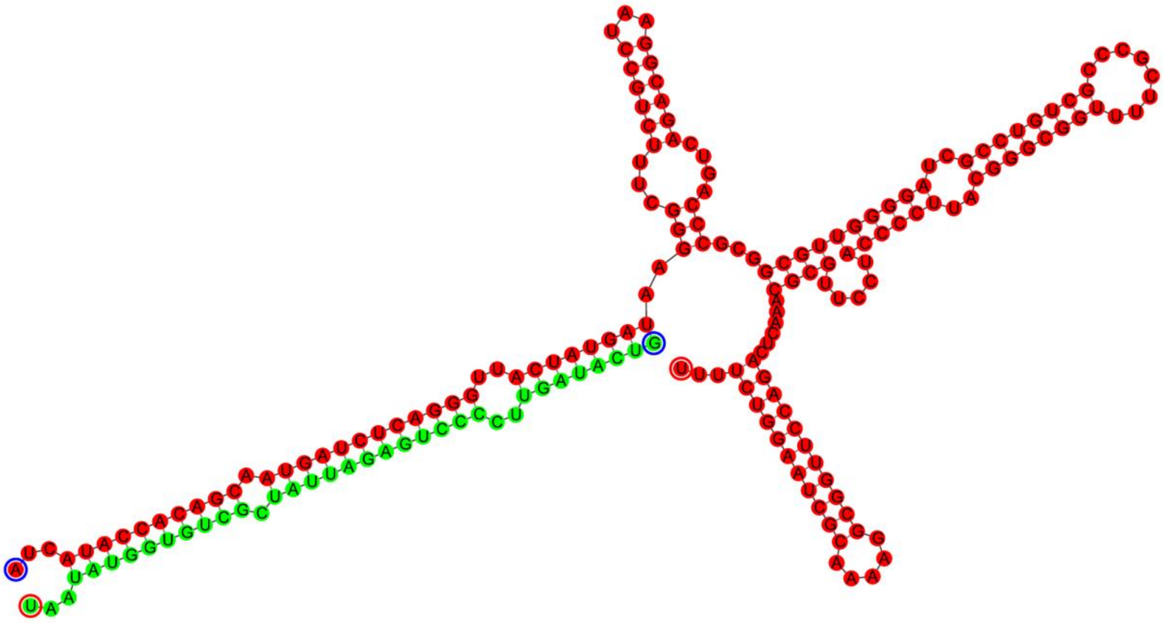

Predicted structure of crRNA:tracrRNA duplex. Sequence in green corresponds to crRNA excluding the spacer, and the sequence in red the tracrRNA sequence. 5ʻ ends are highlighted with blue circle and 3ʻ ends with red circles. Spacer sequence of crRNA is not shown. RNA folding predicted using Geneious 9.1.8.

Supplementary file 5

A:

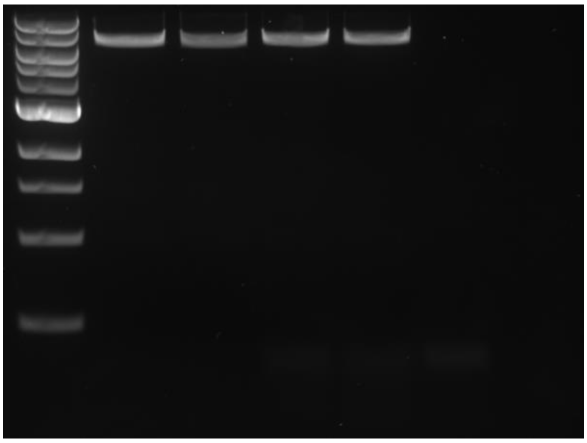

B:

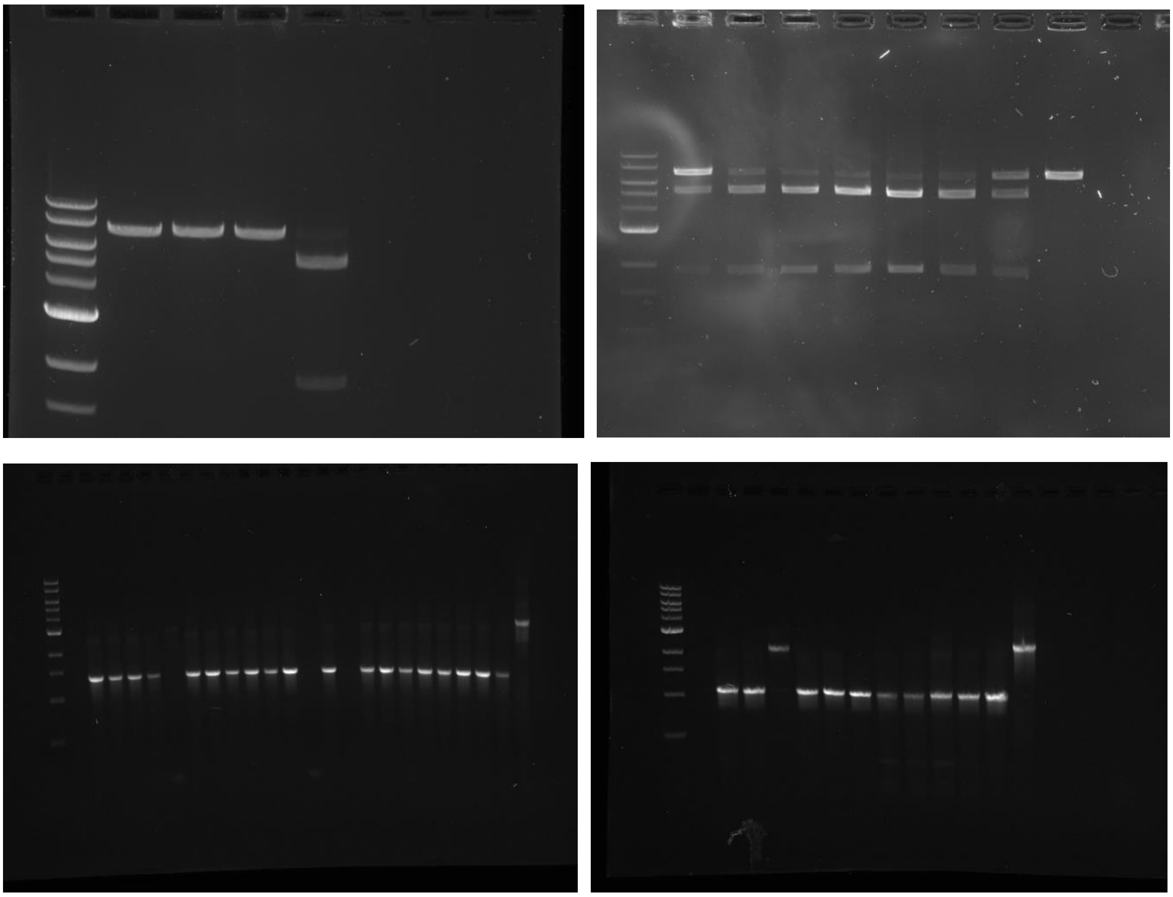

A: ***In vitro* cleavage assay using a non-target gRNA.** CaldoCas9, a DNA ‘target’ and gRNA containing a non-target spacer were incubated in various combinations at 55°C and the products separated on an agarose gel. Samples were loaded onto gel in the same sequence as indicated for figure 2A in the main test, and the ladder sizes are the same.

B: Unprocessed images of agarose gels presented in the main text of the manuscript. In top row, corresponding to Figure 2 in main text, and in bottom row, corresponding to Figure 4 in main text.

Supplementary file 6

A.Frequency in digested library

	A	C	G	T
**1**	14.4	42	19.1	24.5
**2**	52.8	19.1	14.7	13.4
**3**	25.5	32	19.1	23.5
**4**	35.4	14.9	30.7	19.1
**5**	3.3	0.8	95.7	0.2
**6**	35	20.6	8.8	35.6
**7**	66.4	23	5.5	5.1
**8**	89.6	2	4.3	4.1
**9**	25.7	23.8	24.1	26.4
**10**	36.2	20.5	18.2	25.1
**11**	28.7	23.1	25.6	22.6
**12**	31.8	24.8	17.9	25.6

B. Frequency in undigested library

	A	C	G	T
**1**	28.6	19.2	23.1	29.1
**2**	22.7	19.4	24.9	33
**3**	24.1	22.3	21.6	32.1
**4**	23.9	20.8	28.5	26.8
**5**	23.5	21.1	28.6	26.8
**6**	19.4	22.5	23.9	34.3
**7**	19.2	24.1	25	31.7
**8**	24.6	22.5	18.5	34.4
**9**	21.2	24	26.3	28.6
**10**	22.9	18.6	24.9	33.6
**11**	22	23.4	26.9	27.7
**12**	24.6	20.1	26.6	28.7

C. Over/underrepresentation in undigested library (B/25)

	A	C	G	T
**1**	1.14	0.77	0.92	1.16
**2**	0.91	0.78	1.00	1.32
**3**	0.96	0.89	0.86	1.28
**4**	0.96	0.83	1.14	1.07
**5**	0.94	0.84	1.14	1.07
**6**	0.78	0.90	0.96	1.37
**7**	0.77	0.96	1.00	1.27
**8**	0.98	0.90	0.74	1.38
**9**	0.85	0.96	1.05	1.14
**10**	0.92	0.74	1.00	1.34
**11**	0.88	0.94	1.08	1.11
**12**	0.98	0.80	1.06	1.15

D. Digested library normalized for under/overrepresentation (A/C)

	A	C	G	T	Sum
**1**	12.6	54.7	20.7	21.0	109
**2**	58.1	24.6	14.8	10.2	108
**3**	26.5	35.9	22.1	18.3	103
**4**	37.0	17.9	26.9	17.8	100
**5**	3.5	0.9	83.7	0.2	88
**6**	45.1	22.9	9.2	25.9	103
**7**	86.5	23.9	5.5	4.0	120
**8**	91.1	2.2	5.8	3.0	102
**9**	30.3	24.8	22.9	23.1	101
**10**	39.5	27.6	18.3	18.7	104
**11**	32.6	24.7	23.8	20.4	101
**12**	32.3	30.8	16.8	22.3	102

E. Normalized frequency in digested library % (D/Sum)

	A	C	G	T
**1**	12%	50%	19%	19%
**2**	54%	23%	14%	9%
**3**	26%	35%	22%	18%
**4**	37%	18%	27%	18%
**5**	4%	1%	95%	0%
**6**	44%	22%	9%	25%
**7**	72%	20%	5%	3%
**8**	89%	2%	6%	3%
**9**	30%	25%	23%	23%
**10**	38%	26%	18%	18%
**11**	32%	24%	23%	20%
**12**	32%	30%	16%	22%

F. Normalized frequency in digested library relative to most common base

	A	C	G	T
**1**	23%	100%	38%	39%
**2**	100%	42%	25%	17%
**3**	74%	100%	61%	51%
**4**	100%	49%	73%	48%
**5**	4%	1%	100%	0%
**6**	100%	51%	20%	58%
**7**	100%	28%	6%	5%
**8**	100%	2%	6%	3%
**9**	100%	82%	76%	76%
**10**	100%	70%	46%	47%
**11**	100%	76%	73%	63%
**12**	100%	96%	52%	69%

In tables A and B are the observed frequency of bases A, C, G, and T (top row) in nucleotides 1-12 (first column) downstream of the Cas9:gRNA targeted sequence, as revealed by sequencing before (table B) and after (table A) Cas9 digestion. In tables C-F the observed frequency of bases in the digested library are normalized with the frequency in the undigested libray. Calculations are indicated in brackets in the title of tables C-F.

Supplementary file 7

High %GC Codon optimized gene encoding CaldoCas9:

5‘-
ATGCGCTACAAGATCGGCCTGGACATCGGCATCACCAGCGTGGGCTGGGCGGTGATCAACCTCGATATTCCGCGGATCGAGG
ACCTGGGTGTCCGGATTTTCGATCGCGCCGAAAATCCGCAAACGGGAGAAAGCCTGGCCCTGCCCCGTCGGCTGGCGCGCAG
CGCCCGGCGTCGCCTCCGCCGCCGGAAACATCGCCTGGAACGCATTCGCCGTCTGATCATCCGGGAAGGGATCCTGACGAAG
GAGGAGCTGGACAAGCTGTTTGAGGAGAAGCACGAGATCGATGTCTGGCAGCTCCGGGTGGAGGCGCTGGACCGGAAGCTCA
ACAACGACGAGCTGGCGCGGGTCCTGCTGCATCTGGCCAAGCGCCGGGGCTTCAAAAGCAACCGCAAGTCGGAACGCTCGAA
CAAGGAGAACTCGACGATGCTGAAGCACATCGAGGAGAACCGCGCCATCCTGTCCTCGTATCGCACCGTCGGCGAGATGATC
GTGAAAGACCCGAAGTTTGCCCTGCACAAACGTAACAAGGGCGAGAACTACACCAATACCATTGCCCGCGACGACCTGGAGC
GGGAGATCCGGCTCATCTTCTCGAAGCAGCGCGAATTTGGGAACATGTCGTGCACCGAGGAGTTCGAGAACGAATACATTGC
CATCTGGGCCTCGCAGCGGCCGGTGGCGTCGAAGGACGACATTGAGAAGAAGGTGGGGTTCTGCACGTTCGAACCGAAGGAG
AAGCGGGCGCCGAAAGCCACGTACACGTTCCAGTCGTTCATCGCGTGGGAGCACATCAACAAACTCCGGCTGATCTCGCCGT
CGGGGACCCGCGGCCTGACGGATGAGGAACGGCGCCTCCTGTACGAGCAGGCCTTCCAGAAGAACAAGATCACGTACCACGA
CATCCGCACGCTGCTGCACCTGCCGGATGATACGTATTTTAAGGGCATTGTGTATGACCGTGGCGAGTCGCGGAAACAGAAC
GAGAACATCCGGTTCCTGGAGCTCGACGCGTACCACCAAATCCGGAAGGCCGTGGACAAAGTGTACGGCAAAGGCAAATCGT
CCTCCTTCCTGCCGATCGACTTCGATACCTTCGGATATGCCCTGACCCTGTTCAAAGACGACGCGGACATTCGGAGCTATCT
CCGGAACGAATACGAGCAGAACGGTAAACGCATGCCGAACCTGGCCAACAAGGTCTACGACAATGAACTCATCGAAGAACTG
CTCAACCTGTCCTTCACGAAGTTTGGCCACCTGAGCCTGAAGGCCCTGCGCAGCATCCTCCCCTACATGGAGCAGGGCGAAG
TGTACAGCTCGGCCTGTGAGCGCGCGGGATACACCTTCACGGGCCCCAAGAAGAAGCAAAAGACGATGCTGCTGCCGAACAT
TCCGCCCATCGCCAACCCCGTGGTCATGCGGGCCCTGACGCAGGCCCGGAAGGTGGTGAACGCGATCATCAAGAAGTACGGA
AGCCCCGTGTCGATCCACATCGAACTGGCCCGGGACCTGTCGCAGACGTTCGACGAACGCCGCAAAACCAAGAAGGAACAGG
ATGAGAACCGCAAGAAGAACGAAACGGCCATCCGCCAGCTGATGGAGTACGGACTGACGCTGAATCCGACGGGTCACGACAT
CGTGAAATTCAAACTGTGGTCGGAGCAGAACGGCCGCTGCGCCTATTCGCTGCAGCCCATCGAAATCGAGCGGCTCCTGGAA
CCGGGCTACACGGAGGTGGACCACGTCATTCCGTACTCGCGCTCCCTGGATGACTCCTACACCAACAAGGTGCTGGTCCTCA
CGAAGGAGAATCGCGAGAAGGGTAACCGCATCCCGGCCGAGTATCTGGGCGTGGGCACGGAGCGCTGGCAGCAGTTCGAGAC
GTTTGTCCTGACGAACAAACAGTTCAGCAAGAAGAAACGCGACCGGCTGCTGCGCCTGCATTACGACGAAAACGAGGAAACG
GAGTTCAAGAACCGCAACCTGAACGACACGCGCTACATCAGCCGGTTCTTCGCGAACTTCATCCGCGAGCACCTCAAGTTCG
CGGAATCGGACGATAAGCAGAAGGTGTACACCGTCAACGGCCGTGTGACGGCGCATCTGCGCTCGCGCTGGGAGTTCAACAA
GAACCGCGAAGAATCGGATCTGCACCACGCCGTGGACGCCGTGATCGTGGCCTGCACGACCCCGTCGGACATCGCCAAGGTG
ACCGCCTTCTATCAGCGCCGCGAGCAGAACAAGGAACTGGCCAAGAAGACCGAACCGCATTTCCCGCAGCCGTGGCCGCATT
TTGCCGATGAGCTGCGCGCGCGCCTGTCGAAGCACCCGAAAGAATCGATCAAGGCCCTGAACCTGGGGAACTATGACGACCA
GAAGCTGGAGTCCCTGCAGCCCGTGTTCGTCTCCCGGATGCCCAAGCGCAGCGTCACGGGCGCGGCCCATCAGGAAACGCTG
CGCCGCTACGTGGGCATCGACGAACGTTCGGGGAAGATTCAGACGGTGGTGAAAACCAAGCTGAGCGAAATCAAGCTCGATG
CGTCGGGCCACTTTCCCATGTACGGGAAGGAGAGCGATCCCCGTACCTACGAGGCGATCCGGCAGCGCCTGCTGGAACACAA
CAACGACCCCAAGAAAGCCTTTCAGGAGCCCCTCTACAAGCCGAAGAAGAACGGTGAGCCGGGCCCGGTCATTCGGACCGTG
AAGATCATCGACACGAAGAACCAGGTGATTCCGCTGAACGATGGGAAAACGGTGGCCTACAACTCGAATATCGTGCGGGTGG
ACGTGTTCGAGAAGGACGGCAAGTACTACTGCGTCCCCGTCTATACGATGGACATCATGAAGGGCATCCTGCCCAATAAGGC
CATTGAGCCGAACAAGCCGTACAGCGAATGGAAGGAGATGACGGAGGACTACACGTTCCGGTTCTCGCTCTACCCCAACGAT
CTGATCCGCATCGAACTGCCGCGTGAAAAGATCATTAAGACGGCCGGCGGCGAGGAAATCAAAATCAAGGATCTCTTTGCGT
ACTACAAAACCATCCACTCGGGGACCGCCGGCCTGGAGCTGGTCAGCCACGACTGCTCGTTCAGCCTGTCCGGCGTGGGTTC
GCGCACGCTGAAGCGCTTTGAGAAGTACCAGGTGGACGTGCTGGGCAACATCTACAAAGTGCGCGGCGAGAAGCGGGTGGGC
CTGGCGAGCTCGGCCCACAGCAAGACCGGTGAGACGATTCGCCCGCTGCAGTCGACGCGGGACTAG

Supplementary file 8

A

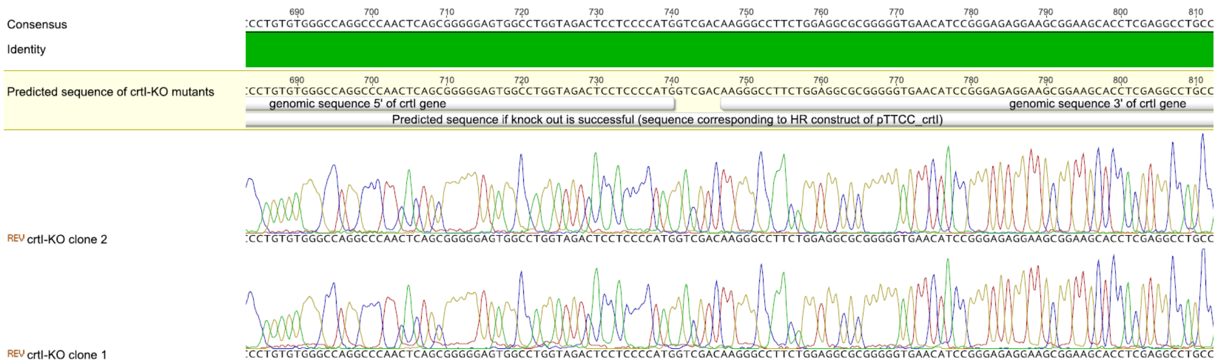

B

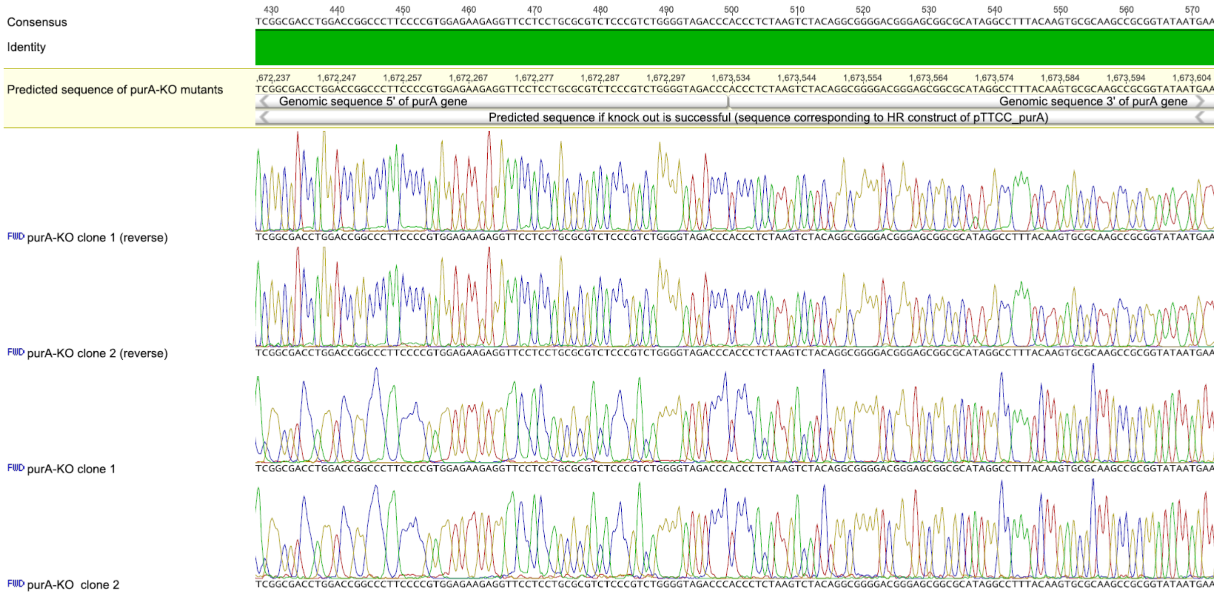

Chromatograms from sequencing of mutant clones, *crtI*-KO (panel A) and *purA*-KO (panel B). The genomic regions surrounding gene *crtI* were amplified with primers F_Tthp_60 and R_Tthp_60, and for *purA* with primers PurA_Verify_F and PurA_Verify_R (the primers are not complimentary to the respective pTTCC genome editing plasmids). The same primers were used for BigDye reactions, performed in duplicate per primer, and the products sequenced. The sequencing data were analyzed in Geneious 9.1.8 software. Ends were trimmed with standard settings (error probability limit 0.01). The trimmed sequences were mapped to a reference consisting of the predicted genomic sequence of KO mutants (track highlighted in yellow color). Results were comparable between the two technical replicates, and therefore the results from only one is shown. Sequencing with primer F_Tthp_60 was not informative because the length of the reads did not span across the edited genomic region.

Supplementary file 9

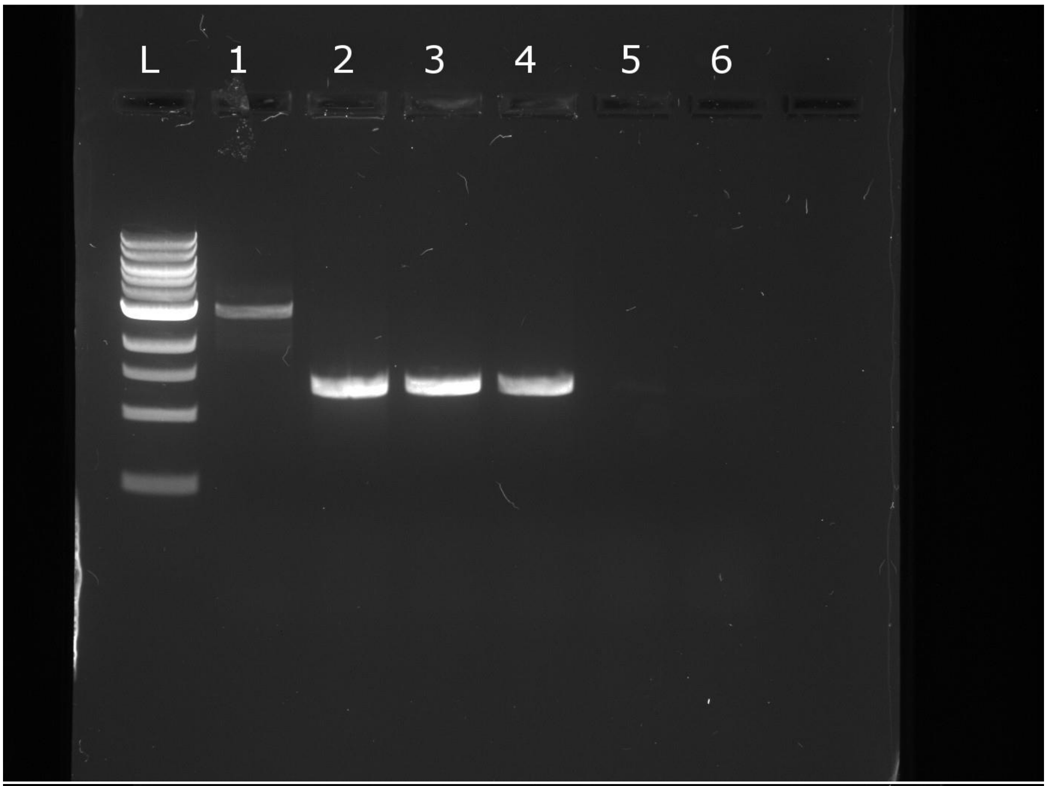

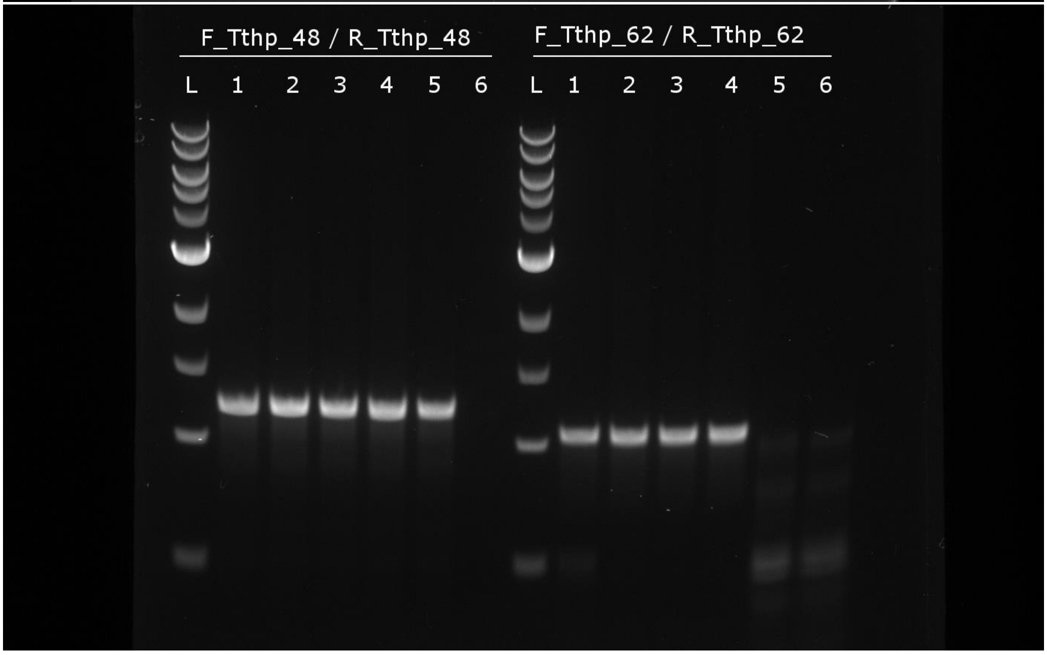

Agarose gel electrophoresis of amplicons after PCR using primer pairs F_Tthp_60 and R_Tthp_60 (top image, expected amplicon size 2891 bp in WT) F_Tthp_48 and R_Tthp_48 (bottom left image; expected amplicon size 1227 bp; amplifies a locus approximately 12 kb upstream of *crtI*), and F_Tthp_62 and R_Tthp_62 (bottom right image; expected amplicon size 1045 bp; amplifies a locus spanning a sequence 0.7-1.7 kb downstream of *crtI*). Sample 1 was WT *T. thermophilus*, samples 2-4 were cells transformed with pTTCC_crtI, and samples 5-6 were cells transformed with pTTCC_crtI_s. Ladder was 1 kb DNA ladder from NEB.

Supplementary file 10

A plasmid map illustrating vector used to express CaldoCas9 in *E. coli*

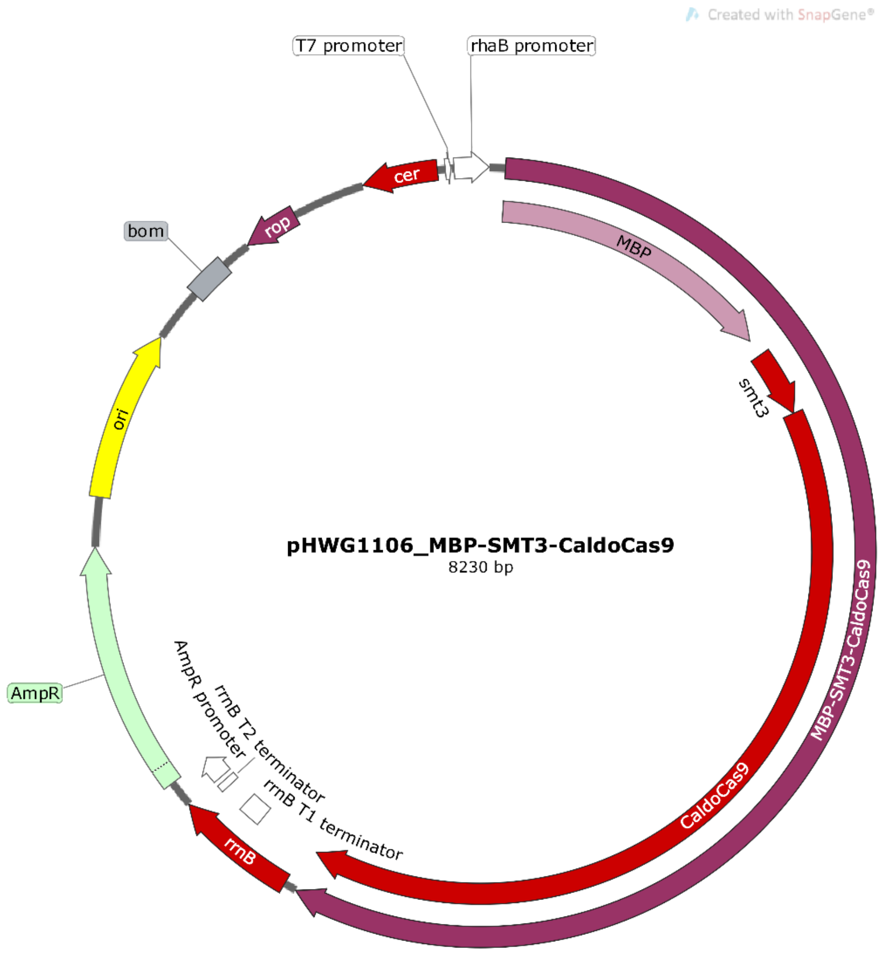

Supplementary file 11

A list of primers used in the study:

**Primer name**	**Primer sequence 5´-**
F_GA_gbCas9Ropt_MalE_pHWH1106	CGTGAACAGATTGGTGGCCGCTACAAGATCGGCCTGG
R_GA_gbCas9Ropt_MalE_pHWH1106	GATATCTTAATGATGATGATGATGATGGCTAGTCCCGCGTCGACTG
R_sgRNA_scaffold	AAAAGACCTTAGCGTTTTCC
MM_gbsgRNA_rtTA_2	CCTCTAATACGACTCACTATAGGGCTAAAGTGCATCTCGGCACCCGCCCAAGTCATAGTTCCCCTGAGATTATCG
GBsgRNAs6	CCTCTAATACGACTCACTATAGGAGTTTTTAGGCCGTAGCGGCTCGAATACGGGTCATAGTTCCCCTGAGATTATCG
gbS6_PAMseq_F	AAGGATCCCCGGGTACCGAGCTGCTAGTTTTTAGGCCGTAGCGGCTCGAATACG
gbS6_PAMseq_pos1to4_R	TGACCATGATTACGAATTCGTTGTTTCNNNNCCGTATTCGAGCCGCTACG
gbS6_PAMseq_pos5to8_R	TGACCATGATTACGAATTCGTTGNNNNTCTGCCGTATTCGAGCCGCTACG
gbS6_PAMseq_pos9to12_R	TGACCATGATTACGAATTCNNNNTTTCTCTGCCGTATTCGAGCCGCTACG
TK-117	CGGCATTCCTGCTGAACCGCTCTTCCGATCT
TK-111*	GATCGGAAGAGCGGTTCAGCAGGAATGCCG
pUC-dir**	GCCAGGGTTTTCCCAGTCACGA
TK-117_seq	gtctcgtgggctcggagatgtgtataagagacagCGGCATTCCTGCTGAACCGCTCTTCCGATCT
pUC-dir_seq	tcgtcggcagcgtcagatgtgtataagagacagGCCAGGGTTTTCCCAGTCACGA
R_gblib_PAMverif**	GTACCGAGCTGCTAGTTTTTAGGC
F_pLEI250_delBbsI	ATGGGACGACCGGGCCCCCTGGGGGCCCAA
R_pLEI250_delBbsI	GCCCGGTCGTCCCATTGCCCGGCATTATAC
F_gbCas9R_NdeI	GGGAATTCCATATGCGCTACAAGATCGGCCTGGAC
R_gbCas9R_HindIII	CCGGGAAGCTTCTAGTCCCGCGTCGACTGCAGC
Q5SDM_4/13_F	AACTGGCCAAAAAGACCGAAC
Q5SDM_4/13_R	CCTTGTTCTGCTCGCGGC
pLEIseqR_trcgRNA	GTGAGAGGTGGGAGAAG
crtI_TT_s3_F	TGGACACGGCCCGGCTCATCGCCCACGACCTGGG
crtI_TT_s3_R2	TGACCCCAGGTCGTGGGCGATGAGCCGGGCCGTG
F2_GA_crtI3end	CCCCATGGTCGACAAGGGCCTTCTGGAGGCGC
R2_GA_crtI3end	CGGAATTCGGTGGTACCGCCTCCTGCAGGGG
F2_GA_crtI5end	CGCTAAGGTCTTTTGGTACCACCAACGTGGTCTCCGAG
R2_GA_crtI5end	GGCCCTTGTCGACCATGGGGAGGAGTCTACCAG
PurA_5F	AACGCTAAGGTCTTTTGGTACCACCCGACGCGGAGAGCCAG
PurA_5R	TCCCGTCTGGGGTAGACCCACCCTCTAAGTCTACAGGC
PurA_3F	CCTGTAGACTTAGAGGGTGGGTCTACCCCAGACGGGAG
PurA_3R	CTCGGAATTCGGTGGTACTCCCGGACGAGAGCGTCCAC
pTTCC_HR_amp_F	CTGTCGCCCGCTTTTGG
pTTCC_HR_amp_R	TCCTGCGTTATCCCCTGATTCT
PurA_Spacer_2_F	TGGACGTGGTCCTCTTCTCCACGAGCCCCAGGCG
PurA_Spacer_2_R	TGACCGCCTGGGGCTCGTGGAGAAGAGGACCACG
F_Tthp_60	CCCACTCCAAGAGGCCAAAG
R_Tthp_60	CCCACCTCCTTCACCATCAC
PurA_Verify_F	TCGCCACCCTCACCTTCCAG
PurA_Verify_R	ACCGCAAGGTCCAGGTCGTC
bgal_amp_F	CACTGGGACTTCGCCCTAAG
bgal_amp_R	GTGAGGAGGTCCCAAAAGGG
crtI_TT_s3_F	TGGACACGGCCCGGCTCATCGCCCACGACCTGGG
F_Tthp_48	TTCTCGGGAAGCTTCACCAC
R_Tthp_48	GCCCGTATCTTGTCCACGAT
F_Tthp_62	GTGATGGTGAAGGAGGTGGG
R_Tthp_62	CTTCCTGGATCTCCTTGCGG

*5’ phosphorylated

**Indicated as P1 and P2 in Figure 2C in main text

Supplementary file 12

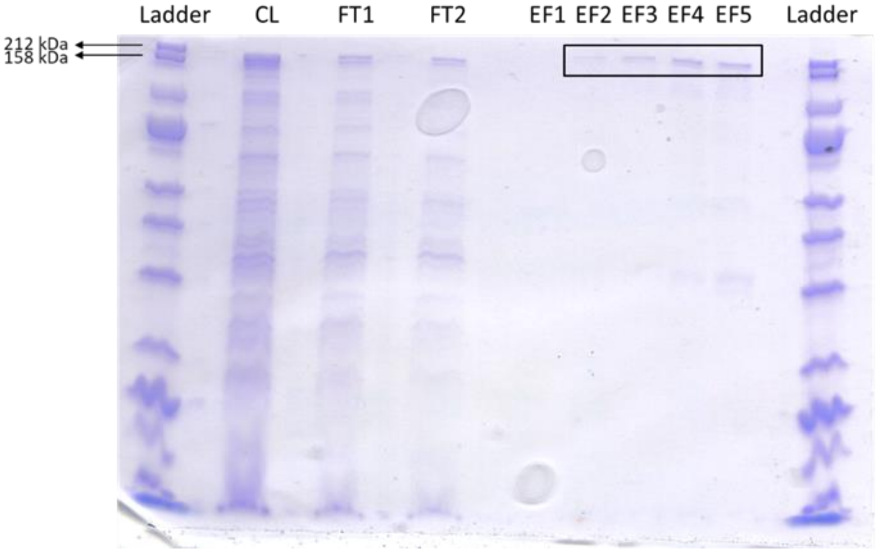

SDS-PAGE on fractions from purification of CaldoCas9-MBP fusion protein on amylose column. CaldoCas9-MBP fusion protein is ~179,2 kDa, indicated for eluted fractions with a box. Cl=Raw cell lysate; FT=Flow-through fractions 1 and 2; EF: Eluted fractions 1, 2, 3, 4 and 5.

Supplementary file 13

A CaldoCas9 gRNA scaffold sequence used to generate gRNA molecules *in vitro:*

5’-
GTCATAGTTCCCCTGAGATTATCGCTGTGGTATAATATCATACCACAGCAATGATCTCAGGGTTACTATGATAAG
GGCTTTCTGCCTAAGGCAGACTGACCCGCGGCGTTGGGGATCGCCTGTCGCCCGCTTTTGGCGGGCATTCCCCAT
CCTTGCGCAAACTCAGACCTTGGCGGAAAACGCTAAGGTCTTTT

Supplementary file 14

A DNA fragment used to clone the gRNA construct to generate pTTCC

5’-
CTGGTCCCGGGGGTACTGAGCTGTTGACAATTAATCATCCGGCTCGTATAATGTGTGGAACGTCTTCTAAGGA
GGTCACATATGACCATGATTACGGATTCACTGGCCGTCGTTTTACAACGTCGTGACTGGGAAAACCCTGGCGTT
ACCCAACTTAATCGCCTTGCAGCACATCCCCCTTTCGCCAGCTGGCGTAATAGCGAAGAGGCCCGCACCGATCG
CCCTTCCCAACAGTTGCGCAGCCTGAATGGCGAATAGGAAGACTAGTCATAGTTCCCCTGAGATTATCGCTGTG
GTATAATATCATACCACAGCAATGATCTCAGGGTTACTATGATAAGGGCTTTCTGCCTAAGGCAGACTGACCCG
CGGCGTTGGGGATCGCCTGTCGCCCGCTTTTGGCGGGCATTCCCCATCCTTGCGCAAACTCAGACCTTGGCGG
AAAACGCTAAGGTCTTTTGGTACCACCGAATTCCG

Supplementary file 15

Protocol for cloning spacers into pTTCC*

Protocol overview:

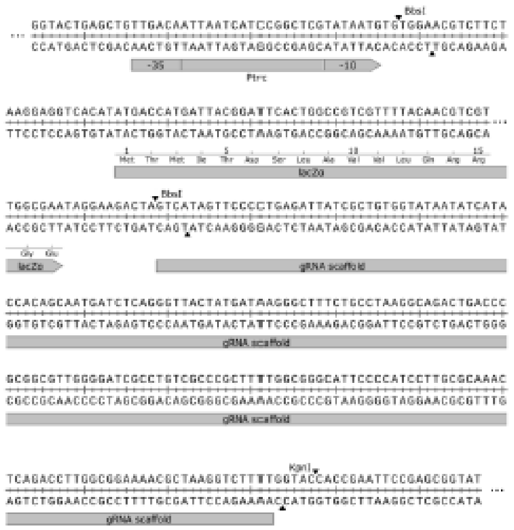

1. Design oligonucleotides containing a desired spacer sequence. Add 4bp overhangs complimentary to BbsI restricted vector pTTCC:

   5'-TGGANNNNNNNNNNNNNNNNNNNNNNNNNNNNNN -3'
   3'- NNNNNNNNNNNNNNNNNNNNNNNNNNNNNNCAGT-5'

2. Anneal and phosphorylate oligos
3. Digest pTTCC with BbsI
4. Ligate annealed and phosphorylated oligos into BbsI restricted pTTCC
5. Transform ligation mix into NEBStable competent cells; culture O/N on L media agar plates with 30 µg/ml kanamycin
6. Culture a few clones in liquid media O/N in L+ Kan (30 µg/ml), miniprep, and confirm spacer insert by restriction and/or sequencing

Protocol details:

1. Design oligonucleotides containing a desired spacer sequence adjacent to a PAM.

   The top-strand oligo should contain an overhang 5'-TGGA followed by the 5'-3' spacer sequence; The bottom-strand oligo should contain an overhang 5'-TGAC followed by the 5'-3' reverse complement of the spacer sequence; gRNAs tested in the present study contained a 30 nt spacer sequence. Other lengths may be suitable but have not been tested.

2. Anneal and phosphorylate the oligos:

   Mix:
   1 µl top-strand oligo (10 µM)
   1 µl bottom-strand oligo (10 µM)
   2 µl T4 DNA ligase buffer (M0202 NEB)
   0.5 µl T4 PNK (M0236 NEB)
   15.5 µl H_2O

   Incubate mix:
   37°C for 60 minutes
   95°C for 5 minutes
   Cool to 10°C in steps, at a rate of -5°C/min

3. Digest pTTCC

   Mix:
   1 µg pTTCC
   1 µl BbsI-HF (NEB R3539)
   5 µl CutSmart buffer (NEB)
   H_2O to 50 µl

   Incubate mix:
   37°C for 1 hour
   65°C for 20 minutes

   Purification of the vector is not neccessary, but may reduce background in the following steps

4. Ligate vector and oligos

   Mix:
   10-40 ng digested vector (step 3)
   1 µl annealed oligos (step 2)
   1 µl 10X T4 DNA ligase buffer (NEB)
   0.5 µl T4 DNA ligase
   H_2O to 10 µl

   Incubate mix:
   24°C for 10 minutes

5. Transform NEBStable (C3040, NEB) according to manufacturer's instructions; plate on L agar media with 30 µg/ml kanamycin.

*The protocol is adapted from instructions for spacer cloning into vector MLM3636 (addgene 43860) from the Joung lab (J. Keith Joung, Harvard Medical School)
